# Notch2 with retinoic acid license IL-23 expression by intestinal EpCAM^+^ DCIR2^+^ cDC2s in mice

**DOI:** 10.1084/jem.20230923

**Published:** 2024-01-05

**Authors:** Daiya Ohara, Yusuke Takeuchi, Hitomi Watanabe, Yoonha Lee, Hiroki Mukoyama, Toshiaki Ohteki, Gen Kondoh, Keiji Hirota

**Affiliations:** 1 Laboratory of Integrative Biological Science, Institute for Life and Medical Sciences, Kyoto University, Kyoto, Japan; 2 Department of Biodefense Research, Medical Research Institute, Tokyo Medical and Dental University, Tokyo, Japan

## Abstract

Despite the importance of IL-23 in mucosal host defense and disease pathogenesis, the mechanisms regulating the development of IL-23–producing mononuclear phagocytes remain poorly understood. Here, we employed an *Il23a*^Venus^ reporter strain to investigate the developmental identity and functional regulation of IL-23–producing cells. We showed that flagellin stimulation or *Citrobacter rodentium* infection led to robust induction of IL-23–producing EpCAM^+^ DCIR2^+^ CD103^−^ cDC2s, termed cDC_IL23,_ which was confined to gut-associated lymphoid tissues, including the mesenteric lymph nodes, cryptopatches, and isolated lymphoid follicles. Furthermore, we demonstrated that Notch2 signaling was crucial for the development of EpCAM^+^ DCIR2^+^ cDC2s, and the combination of Notch2 signaling with retinoic acid signaling controlled their terminal differentiation into cDC_IL23_, supporting a two-step model for the development of gut cDC_IL23_. Our findings provide fundamental insights into the developmental pathways and cellular dynamics of IL-23–producing cDC2s at steady state and during pathogen infection.

## Teaser

An *Il23a*^Venus^ strain reveals the EpCAM^+^ DCIR2^+^ cDC2s as a key cellular source of IL-23 at steady state and during infection with *Citrobacter rodentium*. Combined Notch2 and retinoic acid signals control the development of IL-23–producing cDC2s within gut-associated lymphoid tissues.

## Introduction

Interleukin-23 (IL-23) is vital for the activation of effector functions in group 3 innate lymphoid cells (ILC3s) and IL-17–producing T helper (Th17) cells, which have a pivotal role in gut homeostasis, but have also been implicated in the pathogenesis of inflammatory bowel diseases (Maloy and Kullberg, 2008; Zeng et al., 2019). Notably, IL-22, a downstream target of IL-23, acts on gut epithelial cells to induce the production of antimicrobial peptides and chemokines and promotes tissue regeneration (Keir et al., 2020). Under physiological conditions, the IL-23–IL-22 axis is essential for shaping the normal gut microbiota because it prevents the aberrant expansion of potentially harmful bacterial species (Shih et al., 2014). Conversely, dysregulation of this axis in *Il23a*^−/−^ and *Il22*^−/−^ mice leads to high susceptibility to infection with pathogenic bacteria such as *Citrobacter rodentium* (*C. rodentium*), a model bacterium for human enteropathogenic and enterohemorrhagic *Escherichia coli* infection (Mangan et al., 2006; Zheng et al., 2008). Thus, understanding the regulation of IL-23 is important not only for maintaining gut homeostasis but also for preventing gut infectious diseases. However, the cellular sources of IL-23 in the gut remain controversial (Eken and Oukka, 2016; Luciani et al., 2022).

Accumulating evidence suggests that a subset of mononuclear phagocytes (MNPs), comprising macrophages or conventional dendritic cells (cDCs), which include cDC1 and cDC2 subsets, are a key source of IL-23, necessary to regulate antimicrobial responses to *C. rodentium* (Satpathy et al., 2013; Longman et al., 2014; Aychek et al., 2015). However, the highly heterogeneous nature of gut MNPs poses a challenge in identifying the specific cells that produce IL-23 in response to pathogen infection. Moreover, the underlying mechanisms that regulate the development and function of specific IL-23–producing MNPs in host defense remain incompletely understood. Thus, generating a mouse strain that faithfully reports IL-23 expression would be instrumental for identifying IL-23–producing MNPs and determining the factors necessary for their development, differentiation, and functions.

Here, we developed an Il23a-Venus reporter strain (*Il23a*^Venus^ mice) to allow us to visualize and characterize IL-23–producing MNPs in the gut. By using *Il23a*^Venus^ mice, we identified EpCAM^+^ DCIR2^+^ CD103^−^ CD11b^−^ cDC2s as the primary source of IL-23 at steady state and after *C. rodentium* or flagellin challenge. These IL-23–producing cDC2s with a distinct transcriptional profile were localized to gut-associated lymphoid tissues (GALTs) but not lamina propria tissues of villi or crypts, where large numbers of gut cDCs are located. Notably, Notch2 signaling was found to be key for the development of EpCAM^+^ DCIR2^+^ CD103^−^ CD11b^−^ cDC2s, and retinoic acid signaling was required for the terminal differentiation of these cells into the IL-23–producing EpCAM^+^ DCIR2^+^ cDC2 population. These findings shed light on the developmental identity and functional regulation of the IL-23–producing cDC2 subset in GALTs.

## Results

### *Il23a*^Venus^ mice enabled the identification of a small population of IL-23**–**producing cDC2s in the gut at steady state

**Figure 1.**
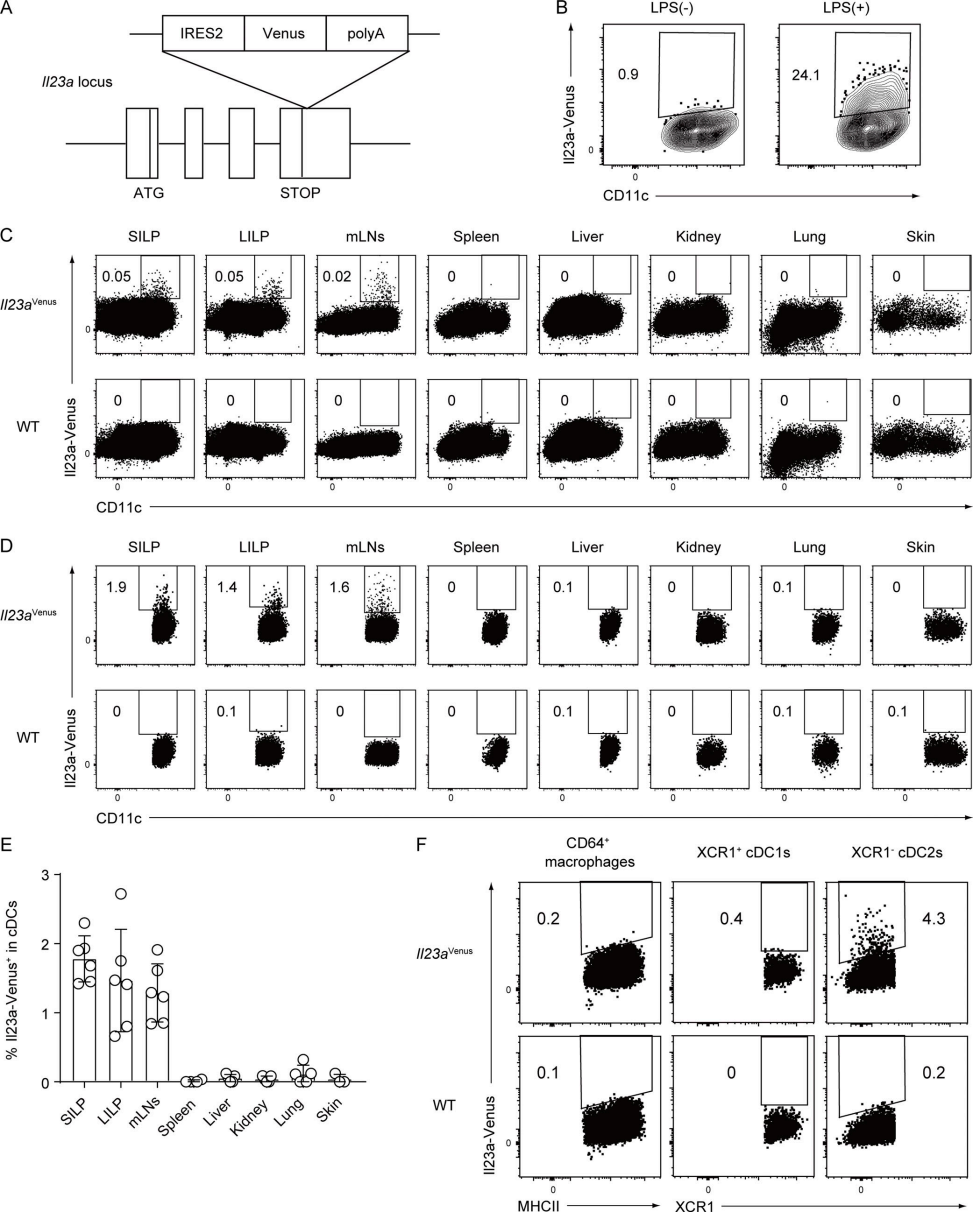
**Generation of an *Il23a***^***Venus***^ **strain and Il23a-Venus expression in cDCs in gut-associated tissues. (A)** Targeted insertion of a Venus reporter gene into the *Il23a* locus. An IRES2-Venus-SV40 late polyA signal cassette was inserted immediately after the *Il23a* translational stop codon, creating a bicistronic locus encoding both *Il23a* and Venus under the control of the *Il23a* promoter. **(B)** The frequency of Il23a-Venus expression in CD11c^+^ bone marrow–derived dendritic cells with or without LPS stimulation. **(C and D)** The frequency of Il23a-Venus expression in CD45^+^ cells (C) or cDC subsets (D) from the SILP, LILP, mLNs, spleen, liver, kidney, lung, and skin of WT or *Il23a*^Venus^ mice at steady state. The gating strategies for cDCs are shown in SourceData F1. **(E)** The percentages of Il23a-Venus^+^ in cDCs from the indicated tissues of *Il23a*^Venus^ mice at steady state (*n* = 3–6). **(F)** The frequency of Il23a-Venus expression in the CD64^+^ macrophages, XCR1^+^ cDC1s, and XCR1^−^ cDC2s from the SILP at steady state. The data in B–D are representative of two independent experiments, and the data in E are pooled from two independent experiments. The data in F are representative of three independent experiments. The graph depicts mean ± SD. Source data are available for this figure: SourceData F1.

To visualize IL-23 expression in MNPs in vivo, we generated an Il23a-Venus reporter strain, which we refer to as *Il23a*^Venus^ mice (Fig. 1 A). The Venus reporter gene is transcribed simultaneously with the endogenous *Il23a* gene under the control of the endogenous *Il23a* promoter. By using *Il23a*^Venus^ mice, we first confirmed the expression of Il23a-Venus in bone marrow–derived dendritic cells, which are known to express IL-23 in response to LPS stimulation (Siegemund et al., 2007; Fig. 1 B). We then investigated Il23a-Venus expression in CD45^+^ immune cells from various tissues at steady state. We found that CD11c^+^ cells in gut-associated tissues like the lamina propria of the small intestine (SILP) and large intestine (LILP), and mesenteric lymph nodes (mLNs) specifically expressed Il23a-Venus at steady state (Fig. 1 C). Gating on cDC subsets, we found that ∼2% of cDCs showed Il23a-Venus expression in SILP, LILP, and mLNs, but not in other organs (Fig. 1, D and E; and SourceData F1). Of note, this flowcytometry analysis of SILP or LILP indicated that Il23a-Venus^+^ cDCs could be located within lamina propria tissues of villi or crypts, or tertiary lymphoid organs. The subsequent flowcytometry analysis for the expression of Il23a-Venus in MNPs such as cDC1s, cDC2s, and macrophages from the SILP showed that the XCR1^−^ cDC2 subset selectively expressed Il23a-Venus, whereas the XCR1^+^ cDC1 subset and macrophages did not (Fig. 1 F). These data indicate that gut cDC2s are the primary source of IL-23 and that the gut microenvironment may be instrumental for the specific upregulation of IL-23 at steady state.

### Il23a-Venus^+^ gut cDCs exhibit high expression of the surface markers EpCAM and DCIR2 at steady state

**Figure 2.**
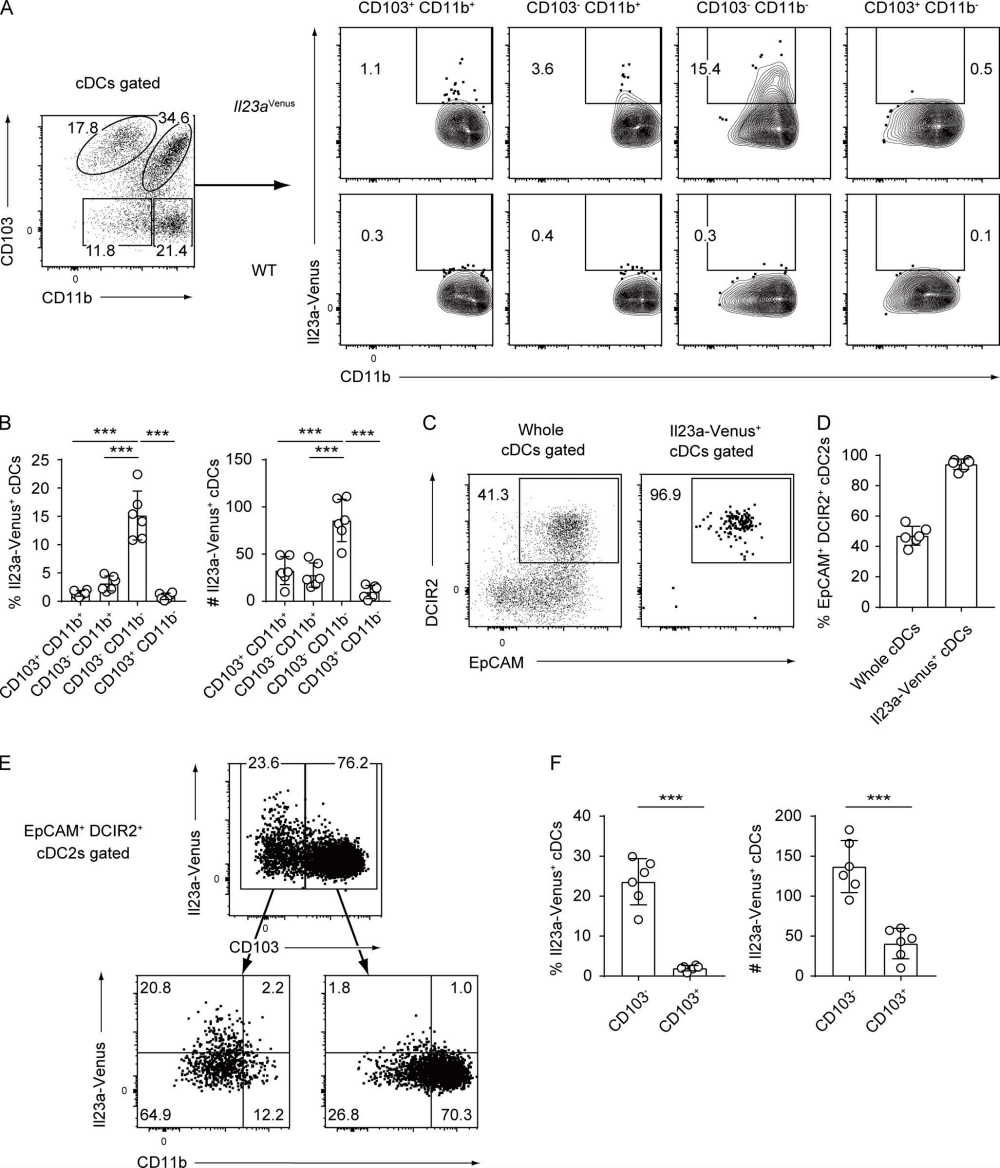
**Il23a-Venus**^**+**^ **gut cDCs exhibit high expression of the surface markers EpCAM and DCIR2 at steady state. (A)** The frequency of Il23a-Venus expression was determined in subpopulations of cDCs (CD103^+^ CD11b^+^, CD103^−^ CD11b^+^, CD103^−^ CD11b^−^, and CD103^+^ CD11b^−^) from WT and *Il23a*^Venus^ mice at steady state. **(B)** The percentages and total cell numbers of Il23a-Venus^+^ cells in the indicated cDC subsets from the SILP at steady state (*n* = 6). **(C)** The frequency of EpCAM^+^ DCIR2^+^ cells in whole cDCs and Il23a-Venus^+^ cDCs from the SILP at steady state. **(D)** The percentages of EpCAM^+^ DCIR2^+^ cells in whole cDCs and Il23a-Venus^+^ cDCs from the SILP (*n* = 6). **(E)** The frequency of Il23a-Venus and CD11b expression by CD103^−^ or CD103^+^ EpCAM^+^ DCIR2^+^ cDCs from the SILP at steady state. **(F)** The percentages and total cell numbers of Il23a-Venus^+^ cells in CD103^−^ or CD103^+^ DCIR2^+^ EpCAM^+^ cDCs from the SILP at steady state (*n* = 6). The data in B, D, and F are pooled from two independent experiments. The data in A, C, and E are representative of three independent experiments. Statistical analyses were performed by one-way ANOVA followed by Tukey’s multiple comparisons test (B) and by Student’s *t* test (F). ***P < 0.001. Graphs depict mean ± SD.

**Figure S1.**
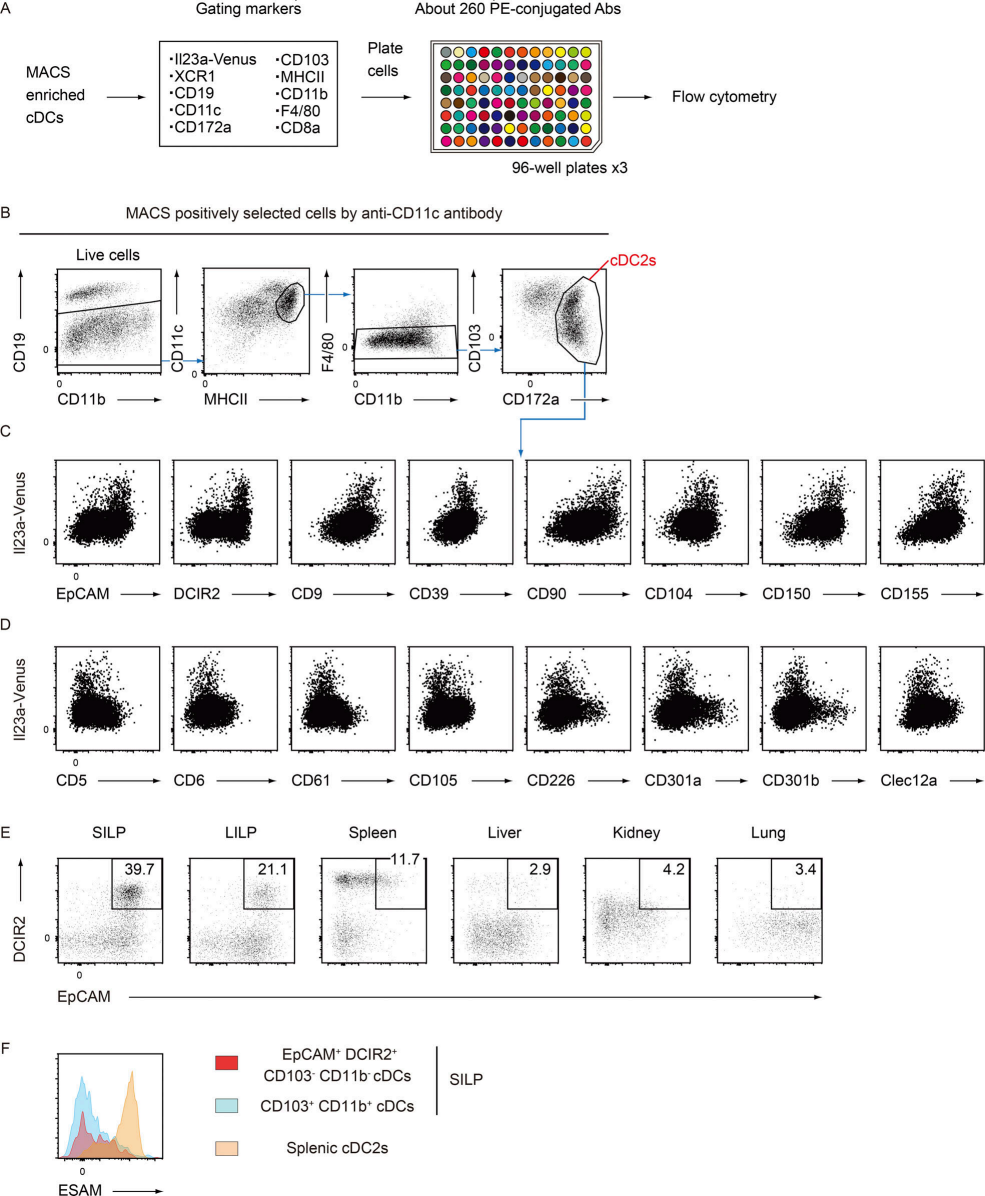
**Screening of surface markers highly associated with Il23a-Venus**^**+**^ **cDCs. (A)** The screening procedure, from cell isolation to flow cytometry analysis. Briefly, CD11c^+^ cells from mLNs of *Il23a*^Venus^ mice were enriched using the MACS system, followed by staining with “gating markers” to define the cDC2 subset. Then, the stained cells were aliquoted across ∼260 wells, each containing a PE-conjugated antibody against a different cell surface protein and analyzed by flow cytometry. **(B)** The gating strategy used to define the cDC2 subset. The subset was gated as CD19^−^ CD11c^+^ MHCII^+^ F4/80^−^ CD172a^+^ cells. Then, the expression of each surface marker was assessed in terms of its correlation with that of Il23a-Venus. **(C and D)** The expression patterns of Il23a-Venus and surface markers of interest in the cDC2 subset. The surface markers that exhibited a positive or negative correlation with the expression of Il23a-Venus are presented in C and D, respectively. **(E)** The frequency of EpCAM^+^ DCIR2^+^ cells in cDCs from the indicated tissues at steady state. **(F)** The expression levels of ESAM in EpCAM^+^ DCIR2^+^ CD103^−^ CD11b^−^ and CD103^+^ CD11b^+^ cDCs from SILP and splenic cDC2s. The data in E and F are representative of two independent experiments.

Since CD103^+^ CD11b^+^ cDC2s have been previously reported to be a potential source of IL-23 (Kinnebrew et al., 2012; Satpathy et al., 2013), Il23a-Venus expression was analyzed in four cDC subpopulations based on CD11b and CD103 expression. Intriguingly, CD103^+^ CD11b^+^, CD103^−^ CD11b^+^, and CD103^−^ CD11b^−^ cDC2s had the potential to express IL-23 to some extent, but the majority of IL-23–expressing cDC2s were found in CD103^−^ CD11b^−^ cDC2s (Fig. 2, A and B). These findings prompted further exploration of reliable surface markers highly correlated with IL-23 expression in cDC2s. We screened ∼260 surface antigens that can be detected with commercially available antibodies (Fig. S1, A–D) and found several surface proteins that were positively or negatively correlated with Il23a-Venus expressions in cDC2s (Fig. S1, C and D). Among these candidates, we employed EpCAM and DCIR2 because we observed a discrete population easily defined by costaining with anti-EpCAM and DCIR2 antibodies. While half of SILP cDCs showed surface expression of EpCAM and DCIR2, ∼95% of Il23a-Venus^+^ cDCs in the SILP were in the EpCAM^+^ DCIR2^+^ population (Fig. 2, C and D). The majority of DCIR2^+^ cDCs in gut-associated tissues exhibited coexpression of EpCAM, whereas a fraction of splenic DCIR2^+^ cDCs showed coexpression of EpCAM (Fig. S1 E). Finally, the combination of CD103 and CD11b expression with EpCAM and DCIR2 expression allowed for the identification of EpCAM^+^ DCIR2^+^ CD103^−^ CD11b^−^ cDC2s, which were highly enriched for IL-23 expression in the gut (Fig. 2, E and F). The EpCAM^+^ DCIR2^+^ CD103^−^ CD11b^−^ cDC2s showed low expression of ESAM, whereas the majority of splenic cDC2s had high expression of ESAM (Fig. S1 F). These markers can be utilized for efficient enrichment of IL-23–producing cDC2s without the need for a reporter system and provide insight into the phenotypic heterogeneity of gut cDC2s.

### Upregulation of IL-23 expression in EpCAM^+^ DCIR2^+^ CD103^−^ CD11b^−^ cDCs after flagellin stimulation

**Figure 3.**
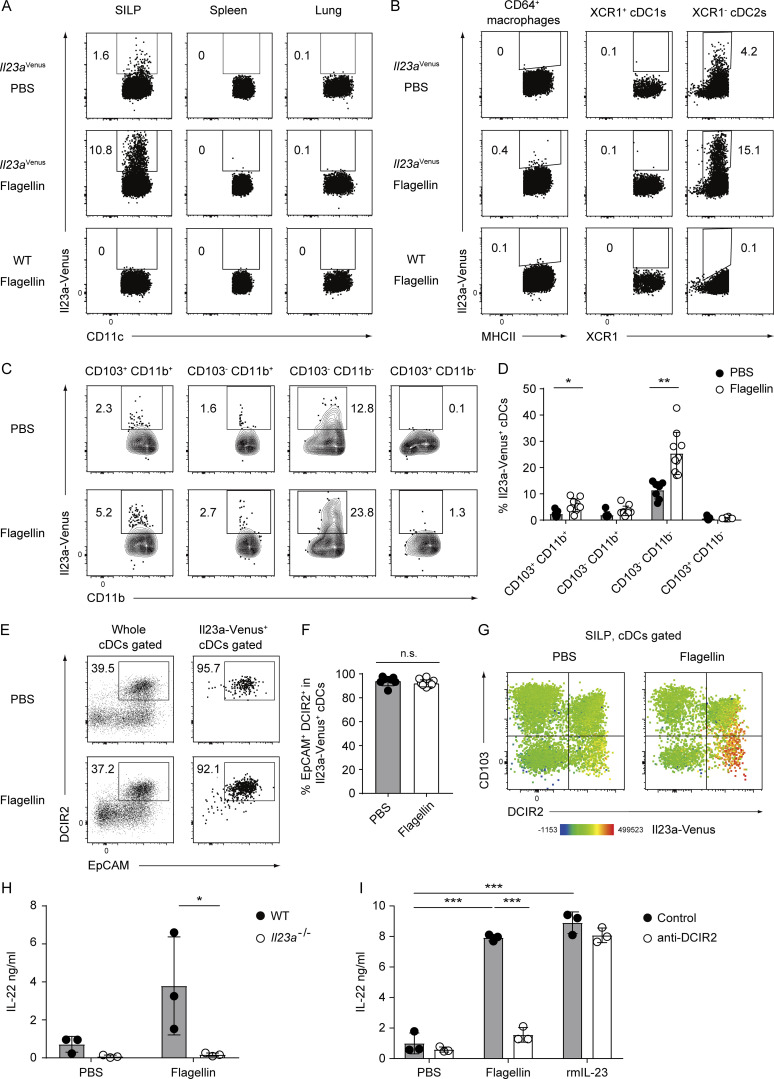
**The administration of flagellin leads to robust induction of IL-23 in the EpCAM**^**+**^ **DCIR2**^**+**^ **CD103**^**−**^ **cDC2s in the gut. (A)** The frequency of Il23a-Venus expression in cDCs from the indicated tissues of WT and *Il23a*^Venus^ mice 4 h after i.v. injection of PBS or flagellin. **(B)** The frequency of Il23a-Venus expression in CD64^+^ macrophages, XCR1^+^ cDC1s, and XCR1^−^ cDC2s from the SILP from WT and *Il23a*^Venus^ mice. **(C)** The frequency of Il23a-Venus expression in subpopulations of cDCs (CD103^+^ CD11b^+^, CD103^−^ CD11b^+^, CD103^−^ CD11b^−^, and CD103^+^ CD11b^−^) from the SILP of *Il23a*^*Venus*^ mice. **(D)** The percentages of Il23a-Venus^+^ cells in the indicated cDC subpopulations (*n* = 7–10). **(E)** The frequency of EpCAM^+^ DCIR2^+^ cells in whole cDCs and Il23a-Venus^+^ cDCs from the SILP. **(F)** The percentages of EpCAM^+^ DCIR2^+^ cells in Il23a-Venus^+^ cDCs (*n* = 7–10). **(G)** The expression of DCIR2, CD103, and Il23a-Venus in SILP cDCs. The heatmap displays a projection of Il23a-Venus expression onto all SILP cDCs. **(H)** IL-22 production by single-cell suspensions of the SILP. Single-cell suspensions from WT and *Il23a*^−/−^ mice were stimulated with flagellin for 16 h, and IL-22 supernatant was measured by ELISA (*n* = 3). **(I)** IL-22 production by the single-cell suspensions of the SILP. Single-cell suspensions from WT mice were stained with an isotype control or anti-DCIR2 antibody, and subsequently, DCIR2^+^ cells were depleted using the MACS system. The collected cells were restimulated with PBS, flagellin, or recombinant mouse IL-23 (rmIL-23) in vitro, and IL-22 supernatant was measured by ELISA (*n* = 3). The data in H and I are representative of two independent experiments. The data in A–C, E, and G are representative of three independent experiments, and the data in D and F are pooled from three independent experiments. Statistical analyses were performed by multiple *t* test comparing PBS- and flagellin-treated groups (D), by Student’s *t* test (F), and by two-way ANOVA followed by Tukey’s multiple comparisons test (H and I). *P < 0.05, **P < 0.01, ***P < 0.001. n.s., not significant. Graphs depict mean ± SD.

**Figure S2.**
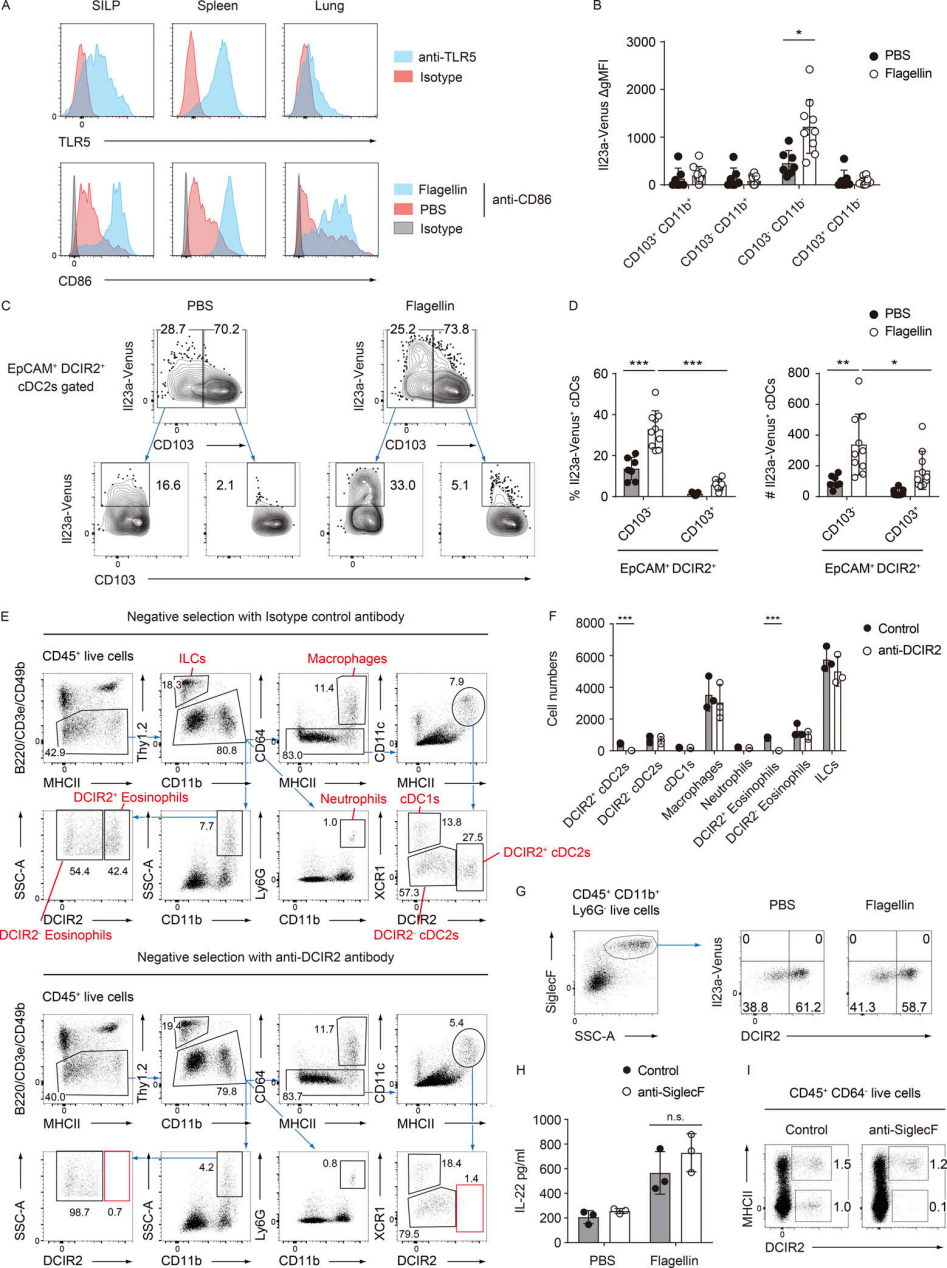
**The EpCAM**^**+**^ **DCIR2**^**+**^ **CD103**^**−**^ **CD11b**^**−**^ **cDC2s are the primary source of IL-23. (A)** The expression levels of TLR5 in cDC2 subsets from the indicated tissues at steady state (upper panel). The expression levels of CD86 in cDC2 subsets from the indicated tissues 4 h after i.v. injection of PBS or flagellin (lower panel). **(B)** The ΔgMFI of Il23a-Venus were determined in subpopulations of cDCs (CD103^+^ CD11b^+^, CD103^−^ CD11b^+^, CD103^−^ CD11b^−^, and CD103^+^ CD11b^−^) 4 h after i.v. injection of PBS or flagellin. The ΔgMFI was calculated by subtracting the Il23a-Venus MFI of WT mice from that of Il23a-Venus mice (*n* = 7–10). **(C)** The frequency of Il23a-Venus expression in CD103^−^ or CD103^+^ EpCAM^+^ DCIR2^+^ cDCs from the SILP 4 h after i.v. injection of PBS or flagellin. **(D)** The percentages and total cell numbers of Il23a-Venus^+^ cells in CD103^−^ or CD103^+^ EpCAM^+^ DCIR2^+^ cDCs from the SILP 4 h after i.v. injection of PBS or flagellin (*n* = 7–10). **(E)** The gating strategy for identifying various immune subsets (DCIR2^+^ cDC2s, DCIR2^−^ cDC2s, XCR1^+^ cDC1s, CD64^+^ macrophages, neutrophils, DCIR2^+^ eosinophils, DCIR2^−^ eosinophils, and ILCs) from the SILP after MACS selection with an isotype-control antibody or anti-DCIR2 antibody, related to Fig. 3 I. **(F)** The total cell numbers of the indicated immune subsets from the SILP after MACS negative selection with an isotype-control antibody or anti-DCIR2 antibody. **(G)** The frequency of Il23a-Venus expression in DCIR2^+^ or DCIR2^−^ eosinophils from the SILP of *Il23a*^Venus^ mice 4 h after i.v. injection of PBS or flagellin. **(H)** IL-22 production by the eosinophil-depleted single-cell suspensions of the SILP. Single-cell suspensions from WT mice were stained with an isotype-control or anti-SiglecF antibody, and subsequently, SiglecF^+^ cells were depleted using the MACS system. The collected cells were restimulated with PBS, or flagellin in vitro, and IL-22 supernatant was measured by ELISA (*n* = 3). **(I)** The frequency of DCIR2^+^ MHCII^+^ cDC2s and DCIR2^+^ MHCII^−^ eosinophils from the SILP after the MACS selection. The data in A and E–I are representative of two independent experiments. The data in C are representative of three independent experiments, and the data in B and D are pooled from three independent experiments. Statistical analyses were performed by multiple *t* test (B, F, and H), and by two-way ANOVA followed by Tukey’s multiple comparisons test (D). *P < 0.05, **P < 0.01, ***P < 0.001. Graphs depict mean ± SD.

Given that systemic administration of flagellin, a ligand of TLR5, results in IL-23 production by CD103^+^ CD11b^+^ cDC2s, followed by IL-22 expression in the SILP (Kinnebrew et al., 2012), we administered flagellin to *Il23a*^Venus^ mice to investigate the potential impact of its stimulation on IL-23 expression in tissue cDCs. Following flagellin injection, we observed selective upregulation of Il23a-Venus expression in the SILP cDCs but not in the splenic and lung cDCs (Fig. 3 A). The induction of IL-23 was not attributed to the activation status of cDCs, as splenic and lung cDCs exhibited upregulation of the activation marker CD86 after flagellin injection (Fig. S2 A).

In the SILP, upregulation of IL-23 was restricted to cDC2s, while both the cDC1 subset and CD64^+^ macrophages exhibited limited expression of Il23a-Venus, after flagellin injection (Fig. 3 B). Among the cDC2 subpopulations, the CD103^−^ CD11b^−^ cDC2s responded most strongly to flagellin stimulation, followed by the CD103^+^ CD11b^+^ cDC2s. Notably, the intensity of the Il23a-Venus signal in the CD103^−^ CD11b^−^ cDC2s increased in response to flagellin stimulation (Fig. 3, C and D; and Fig. S2 B). Of note, the vast majority of Il23a-Venus^+^ cDCs exhibited coexpression of EpCAM and DCIR2 after flagellin injection, similar to their phenotype at steady state (Fig. 3, E and F). In particular, the EpCAM^+^ DCIR2^+^ CD103^−^ CD11b^−^ cDC2s were identified as the predominant source of IL-23 after flagellin injection (Fig. 3 G; and Fig. S2, C and D).

To evaluate the potential role of the EpCAM^+^ DCIR2^+^ cDC2s in mediating the gut IL-23–IL-22 axis, we performed in vitro depletion of DCIR2^+^ cells in a SILP single-cell suspension using an anti-DCIR2 antibody and used Il23a^−/−^ cells as control. Upon flagellin stimulation, IL-23–dependent production of IL-22 from the wild-type (WT) SILP suspension was observed and as expected, impaired in the control culture of Il23a^−/−^ cells (Fig. 3 H). In line with the prediction that EpCAM^+^ DCIR2^+^ cDC2s are a key source of IL-23, IL-22 production was diminished in the culture supernatant from the DCIR2^+^ cell-depleted SILP suspension. This effect was restored upon the addition of recombinant IL-23 (Fig. 3 I). Although DCIR2^+^ eosinophils were also depleted in this assay (Fig. S2, E and F), it is unlikely that eosinophils were the cellular source of IL-23, as Il23a-Venus expression was not observed in these cells after flagellin injection (Fig. S2 G), as well as the in vitro depletion of eosinophils using an anti-SiglecF antibody instead of the anti-DCIR2 antibody did not result in any alteration in IL-22 production after flagellin stimulation (Fig. S2, H and I). Collectively, these findings indicate that IL-23 expression in response to TLR5 signaling is restricted to gut cDC2s and that activated IL-23–producing EpCAM^+^ DCIR2^+^ CD103^−^ CD11b^−^ cDC2s drive the IL-23–IL-22 axis in the gut.

### EpCAM^+^ DCIR2^+^ CD103^−^ CD11b^−^ IL-23**–**producing cells are bona fide cDCs

**Figure 4.**
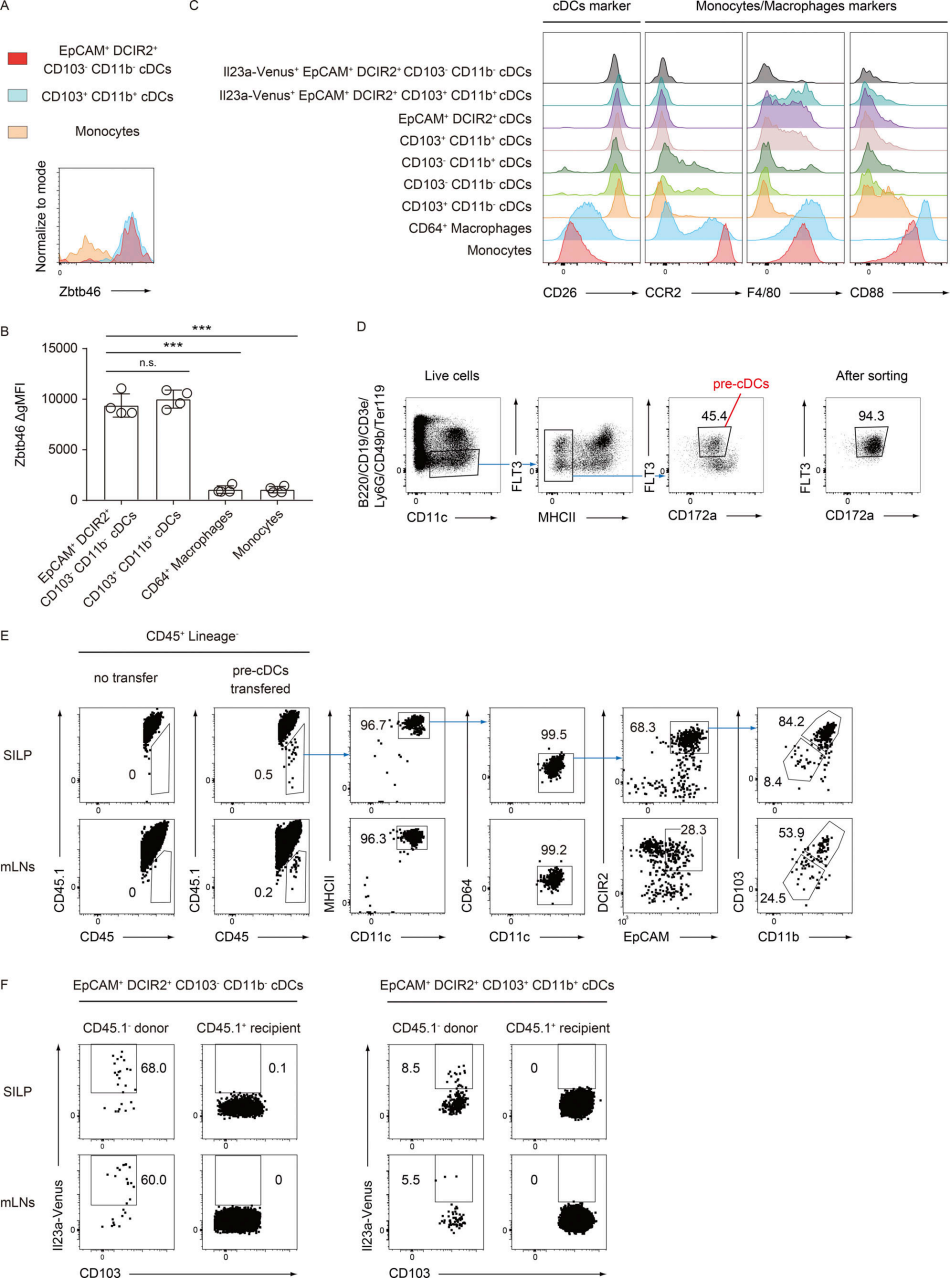
**The development of IL-23–producing EpCAM**^**+**^ **DCIR2**^**+**^ **CD103**^**−**^ **CD11b**^**−**^ **cDCs originates from pre-cDCs. (A)** The expression levels of Zbtb46 in the indicated cell populations isolated from SILP at steady state. **(B)** The delta geometric mean fluorescent intensities (ΔgMFI) of Zbtb46 were determined in the indicated cell populations. The ΔgMFI was calculated by subtracting the Zbtb46 MFI from the control MFI stained with an isotype-control antibody (*n* = 4). **(C)** The expression levels of CD26, CCR2, F4/80, and CD88 in the indicated cell populations isolated from SILP 4 h after i.v. injection of flagellin. **(D)** The gating strategy for sorting pre-cDCs. Lineage^−^ MHCII^−^ CD11c^+^ CD172a^mid^ Flt3^+^ cells were sorted as pre-cDCs from the bone marrow of Il23aVenus mice. The sorting purity was confirmed, as shown in the far-right panel. **(E)** A highly purified population of pre-cDCs from CD45.2^+^ Il23aVenus mice was adoptively transferred into CD45.1^+^ mice. 7 days after transfer, donor-derived CD45^+^ CD3e^−^ CD19^−^ B220^−^ CD49b^−^ Ly6G^−^ SiglecF^−^ cells in SILP and mLNs were analyzed for the indicated markers 4 h after i.v. injection of flagellin. **(F)** The frequency of Il23a-Venus expression in donor-derived or recipient-derived EpCAM^+^ DCIR2^+^ CD103^−^ CD11b^−^ and EpCAM^+^ DCIR2^+^ CD103^+^ CD11b^+^ cDCs from SILP and mLNs. The data in A–F are representative of two independent experiments. Statistical analyses were performed by one-way ANOVA followed by Tukey’s multiple comparisons test in C. ***P < 0.001. n.s., not significant. Graphs depict mean ± SD.

To characterize the developmental origin of the EpCAM^+^ DCIR2^+^ CD103^−^ CD11b^−^ IL-23–producing cells in the gut, we assessed the expression of cDC- and monocyte/macrophage-specific molecules in comparison to other cDC subsets and monocytes/macrophages. EpCAM^+^ DCIR2^+^ CD103^−^ CD11b^−^ cDCs displayed high levels of the cDC-specific transcription factor Zbtb46, akin to CD103^+^ CD11b^+^ cDC2s, which are recognized as part of the cDC lineage, while monocytes and CD64^+^ macrophages did not exhibit Zbtb46 (Satpathy et al., 2012, 2013; Meredith et al., 2012; Fig. 4, A and B). Additionally, we assessed CD26 as a cDC marker and CCR2, F4/80, and CD88 as monocyte/macrophage markers in conjunction with Il23a-Venus expression (Miller et al., 2012). The Il23a-Venus^+^ EpCAM^+^ DCIR2^+^ CD103^−^ CD11b^−^ cDCs showed a similar CD26 expression pattern to CD103^+^ CD11b^−^ cDC1s and CD103^+^ CD11b^+^ cDC2s, whereas they did not express CCR2, F4/80, and CD88 (Fig. 4 C).

We next examined whether the Il23a-Venus^+^ EpCAM^+^ DCIR2^+^ CD103^−^ CD11b^−^ cDCs originated from the precursor of the cDC lineage. To this end, we sorted pre-cDCs defined by lineage^−^ MHCII^−^ CD11c^+^ CD172a^int^ Flt3^+^ cells from the bone marrow of CD45.2^+^ *Il23a*^Venus^ mice and transferred them into CD45.1^+^ CD45.2^+^ mice (Liu et al., 2009; Fig. 4 D). After 7 days, we analyzed CD45.1^−^ donor-derived cells 4 h after flagellin injection. As expected, the vast majority of pre-cDCs differentiated into CD11c^+^ MHCII^+^ CD64^−^ cDCs in SILP and mLN, consisting of both EpCAM^+^ DCIR2^+^ CD103^−^ CD11b^−^ and CD103^+^ CD11b^+^ cells (Fig. 4 E). Furthermore, this adoptive transfer experiment confirmed that the predominant expression of Il23a-Venus was derived from EpCAM^+^ DCIR2^+^ CD103^−^ CD11b^−^ cDCs rather than CD103^+^ CD11b^+^ cDCs (Fig. 4 F). Taken together, these findings establish the identity of the EpCAM^+^ DCIR2^+^ CD103^−^ CD11b^−^ IL-23–producing cells in the gut as a subset within the cDC lineage.

### IL-23**–**producing cDCs are confined to GALTs

**Figure 5.**
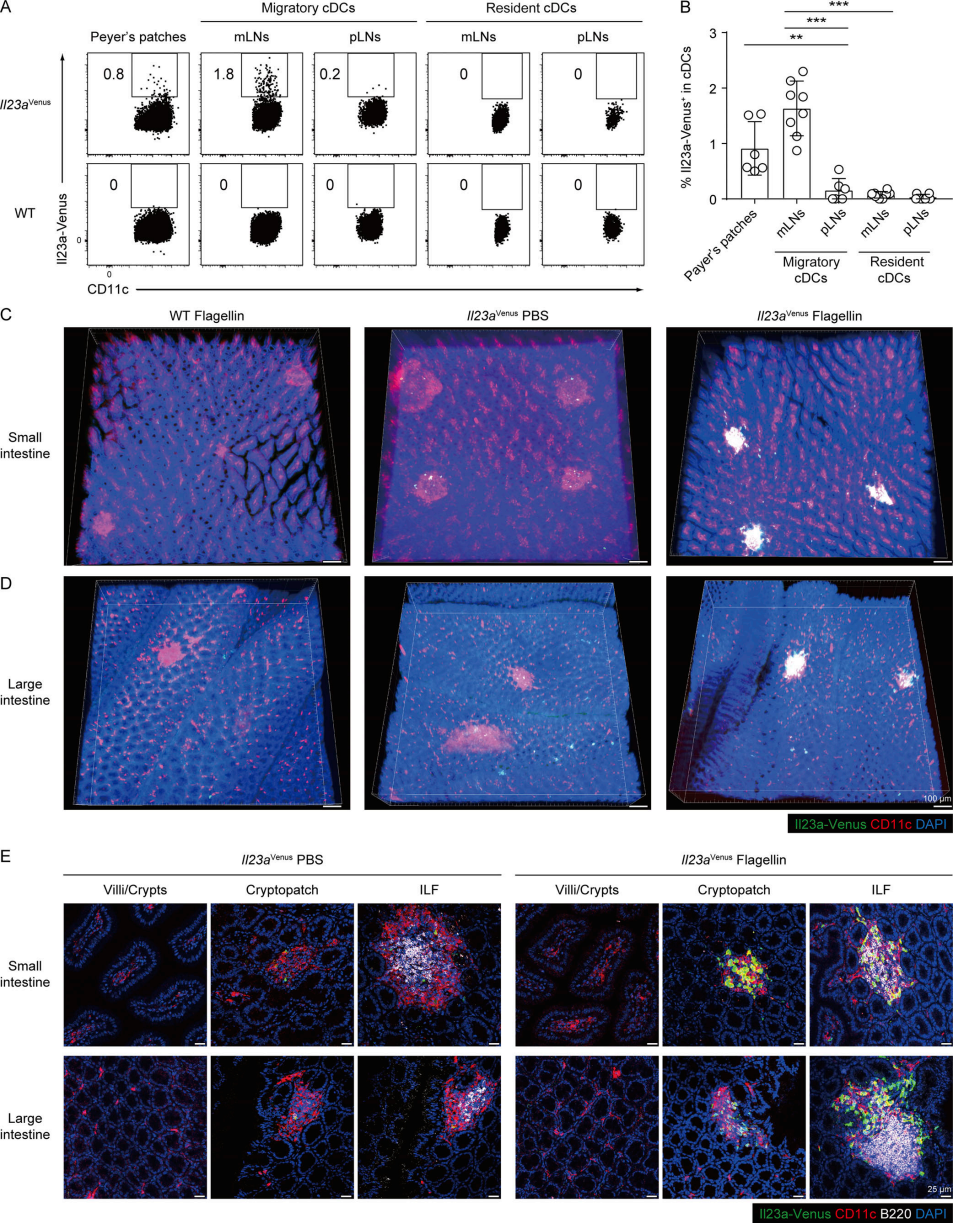
**Il23a-Venus**^**+**^ **cDCs are confined to GALTs. (A)** The frequency of Il23a-Venus expression in cDC subsets isolated from the Peyer’s patches, mLNs, and peripheral LNs (pLNs) of WT and *Il23a*^Venus^ mice at steady state. A gating strategy for separating migratory and resident cDCs is shown in Fig. S3 A. **(B)** The percentages of Il23a-Venus^+^ cells in cDCs from the indicated tissues of *Il23a*^Venus^ mice at steady state (*n* = 6–8). **(C and D)** Whole-mount imaging of the small intestines (C) and large intestines (D) from WT and *Il23a*^Venus^ mice 4 h after i.v. injection of PBS or flagellin. Il23a-Venus, CD11c, and DAPI are depicted in green, red, and blue, respectively. The cells expressing both Il23a-Venus and CD11c are depicted in white. The scale bars indicate a length of 100 μm. **(E)** High-magnification images of selected intestinal structures from optically cleared samples of the small and large intestines of *Il23a*^Venus^ mice. Il23a-Venus, CD11c, B220, and DAPI are depicted in green, red, white, and blue, respectively. The scale bars indicate a length of 25 μm. The data in A and C–E are representative of two independent experiments, and the data in B are pooled from two independent experiments. Statistical analyses were performed by one-way ANOVA followed by Tukey’s multiple comparisons test (B). **P < 0.01, ***P < 0.001. Graphs depict mean ± SD.

**Figure S3.**
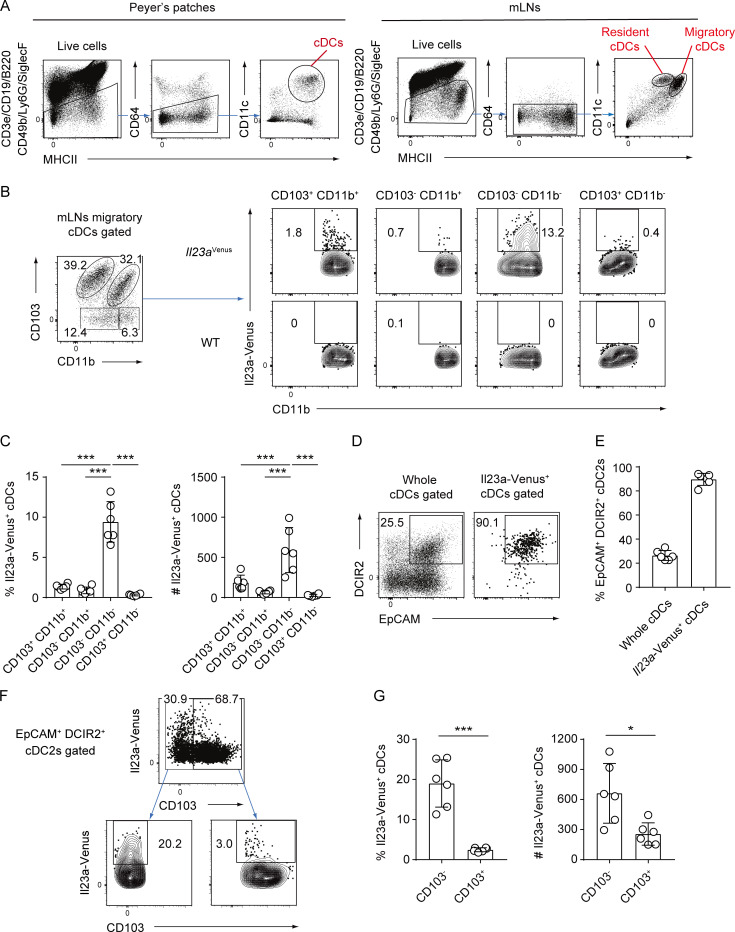
**Migratory cDCs expressing Il23a-Venus in the mLNs show a surface marker expression pattern similar to that of Il23a-Venus**^**+**^ **cDCs in SILP. (A)** The gating strategy for defining cDCs in Peyer’s patches or mLNs. Live CD19^−^ B220^−^ CD49b^−^ Ly6G^−^ SiglecF^−^ CD64^−^ MHCII^+^ CD11c^+^ cells were gated as cDCs. In the mLNs, cDCs were further classified into two cDC subsets: the MHCII^high^ CD11c^+^ migratory and MHCII^+^ CD11c^high^ resident cDCs. **(B)** The frequency of Il23a-Venus expression in different subpopulations of mLN migratory cDCs defined by the expression levels of CD103 and CD11b. The gating strategy used to define the four subpopulations of mLN migratory cDCs was based on the expression levels of CD103 and CD11b. The frequency of Il23a-Venus expression was determined in subpopulations of cDCs (CD103^+^ CD11b^+^, CD103^−^ CD11b^+^, CD103^−^ CD11b^−^, or CD103^+^ CD11b^−^) from WT and *Il23a*^*Venus*^ mice at steady state. **(C)** The percentages and total cell numbers of Il23a-Venus^+^ cells in the indicated migratory cDC subpopulations from the mLN at steady state (*n* = 6). **(D)** The frequency of EpCAM^+^ DCIR2^+^ cells in whole migratory cDCs and Il23a-Venus^+^ cDCs from the mLNs at steady state. **(E)** The percentages of EpCAM^+^ DCIR2^+^ cells in whole migratory cDCs and Il23a-Venus^+^ cDCs from the mLNs (*n* = 6). **(F)** The frequency of Il23a-Venus expression by CD103^−^ or CD103^+^ EpCAM^+^ DCIR2^+^ migratory cDCs from mLNs at steady state. **(G)** The percentages and total cell numbers of Il23a-Venus^+^ cells among CD103^−^ or CD103^+^ EpCAM^+^ DCIR2^+^ migratory cDCs from the mLNs at steady state (*n* = 6). The data in C, E, and G are pooled from two independent experiments. The data in A, B, D, and F are representative of three independent experiments. Statistical analyses were performed by one-way ANOVA followed by Tukey’s multiple comparisons test (C), and by Student’s *t* test (G). *P < 0.05, ***P < 0.001. Graphs depict mean ± SD.

We sought to examine the unique distribution of IL-23–producing cDCs in the gut by utilizing *Il23a*^Venus^ mice. As sentinels of the gut immune system, cDCs are distributed across various gut structures, including lamina propria tissues of villi or crypts, as well as secondary and tertiary lymphoid organs (Luciani et al., 2022). In secondary lymphoid organs, we detected Il23a-Venus expression in ∼1% of cDCs from Peyer’s patches and ∼2% of migratory cDCs but not resident cDCs from mLNs. However, we observed little Il23a-Venus expression in cDCs from peripheral LNs (Fig. 5, A and B; and Fig. S3 A). Consistent with a key feature of IL-23–producing cDCs from the SILP, the CD103^−^ CD11b^−^ cDC2s from the mLNs highly expressed Il23a-Venus with increased intensity, and IL-23–producing cDC2s displayed high levels of EpCAM and DCIR2 (Fig. S3, B–G).

Given the technical limitations in determining the tissue localization of IL-23–producing cDCs by flowcytometry, we next employed clearing-enhanced 3D (Ce3D) clarification procedures to investigate the localization of Il23a-Venus^+^ cDCs in lamina propria tissues of villi or crypts, as well as tertiary lymphoid organs from the small and large intestines (Li et al., 2017). We detected a small number of Il23a-Venus^+^ CD11c^+^ cDCs in the tertiary lymphoid organs such as cryptopatches and isolated lymphoid follicles (ILFs) at steady state. Notably, the number of IL-23–producing CD11c^+^ cDCs was specifically increased in tertiary lymphoid organs following flagellin injection, but not in lamina propria tissues of villi or crypts where many CD11c^+^ cDCs are distributed (Fig. 5, C–E). These results suggest that Il23a-Venus^+^ cDCs are confined to GALTs and that this localization likely enables their close interaction with ILCs in cryptopatches and ILFs to regulate host defense via the IL-23–IL-22 axis (Keir et al., 2020).

### IL-23**–**producing gut cDCs exhibit a distinct transcriptome profile marked by high expression of genes involved in the Notch2 and retinoic acid signaling pathways

**Figure 6.**
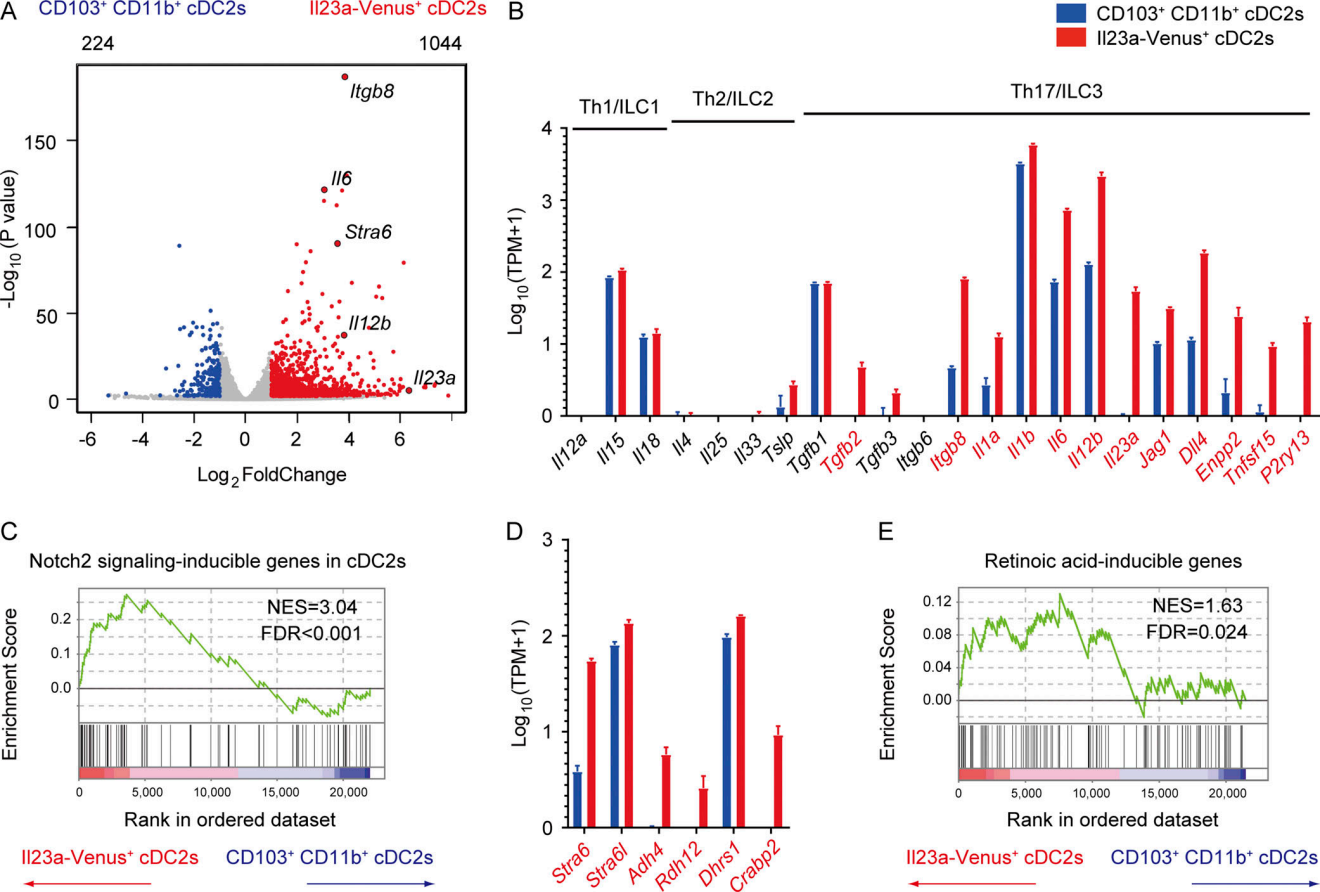
**Il23a-Venus**^**+**^ **cDCs show a distinctive transcriptome profile with high expression of genes involved in the Notch2 and retinoic acid signaling pathways. (A)** Volcano plot showing differentially expressed genes with a Log2foldChange <1 and an adjusted P value <0.05 in the comparison of transcriptome profiles between CD103^+^ CD11b^+^ cDC2s and Il23a-Venus^+^ cDC2s. The upper left and upper right of the plot indicate the total numbers of genes highly expressed in CD103^+^ CD11b^+^ cDC2s and Il23a-Venus^+^ cDC2s, which are represented in blue and red, respectively. **(B)** The expression levels of genes associated with the differentiation and activation of Th1/ILC1, Th2/ILC2, or Th17/ILC3 in the indicated cDC2 subsets (*n* = 3–4). **(C)** GSEA of transcriptome profiles between Il23a-Venus^+^ cDC2s and CD103^+^ CD11b^+^ cDC2s using gene sets induced by Notch2 signaling in cDCs as detailed in the Materials and methods section. **(D)** The expression levels of genes related to vitamin A metabolism in the indicated cDC2 populations (*n* = 3–4). **(E)** GSEA of transcriptome profiles between Il23a-Venus^+^ cDC2s and CD103^+^ CD11b^+^ cDC2s using gene sets induced by retinoic acid as detailed in the Materials and methods section. The three and four individual cDNA libraries from CD103^+^ CD11b^+^ cDC2s and Il23a-Venus^+^ cDC2s were prepared and sequenced. The genes exhibiting significantly higher expression in Il23a-Venus^+^ cDC2s than in CD103^+^ CD11b^+^ cDC2s, as determined by DESeq2 with an adjusted P value <0.05, are depicted in red in panels B and D. The vertical bars in panels B and D denote the SEM. The NES and FDR are shown in panels C and E. Source data are available for this figure: SourceData F6.

To elucidate the molecular basis of IL-23–producing cDCs, we conducted mRNA sequencing (mRNA-seq) analysis to compare Il23a-Venus^+^ cDC2s with CD103^+^ CD11b^+^ cDC2s, which were sorted from the migratory cDCs fraction of mLNs at steady state (SourceData F6). Our differential gene expression analysis revealed that 1,044 genes were upregulated in Il23a-Venus^+^ cDC2s, whereas 224 were downregulated (Fig. 6 A). Notably, Il23a-Venus^+^ cDC2s highly expressed genes associated with the activation of type 17 immunity (McGeachy and Cua, 2008; Zhou and Sonnenberg, 2020). In contrast, the expression levels of genes related to Th1/ILC1 and Th2/ILC2 cells were low or comparable with those of CD103^+^ CD11b^+^ cDC2s (Fig. 6 B). These data suggest a potential role of Il23a-Venus^+^ cDC2s in controlling the differentiation and activation of Th17 cells and ILC3s more potently than CD103^+^ CD11b^+^ cDC2s.

To investigate the potential impact of upstream signaling events on the development, differentiation, and functions of IL-23–producing cDCs, we initially examined the Notch2 signaling pathway. Previous studies have reported the crucial role of Notch2 in regulating the development of the CD103^+^ CD11b^+^ cDC2s, particularly in relation to IL-23 production in the gut (Satpathy et al., 2013; Lewis et al., 2011). Although we observed distinct transcriptome profiles for Il23a-Venus^+^ cDC2s and the CD103^+^ CD11b^+^ cDC2s (Fig. 6 A), we postulated that these populations may share signaling pathways, such as Notch2 signaling, particularly in the context of IL-23 induction. To this end, we conducted gene set enrichment analysis (GSEA; Subramanian et al., 2005) utilizing the top 100 Notch2-dependent genes in the splenic CD11b^+^ cDC2 subset, according to publicly available data (Satpathy et al., 2013). Our analysis revealed that Notch2 signaling-inducible genes were highly enriched in the Il23a-Venus^+^ cDC2s compared with the CD103^+^ CD11b^+^ cDC2s (Fig. 6 C).

We further analyzed genes involved in a signaling pathway related to retinoic acid as an environmental factor that could potentially impact the development or function of IL-23–producing cDCs. We found increased expression of gene modules for retinol uptake (*Stra6* and *Stra6l*), retinoic acid production (*Adh4*, *Rdh12*, and *Dhrs1*), and the retinoic acid’s intracellular carrier protein *Crabp2* in Il23a-Venus^+^ cDC2s (Kam et al., 2012; Napoli, 2016; Fig. 6 D). Furthermore, by using a set of retinoic acid–inducible genes (Balmer and Blomhoff, 2002), we showed by GSEA that retinoic acid–inducible genes were significantly enriched in Il23a-Venus^+^ cDC2s compared with CD103^+^ CD11b^+^ cDC2s (Fig. 6 E). Overall, these observations suggest that Il23a-Venus^+^ cDC2s have a unique gene signature actively receiving signaling pathways of Notch2 and retinoic acid, which could be crucial for the development or function of gut IL-23–producing cDCs.

### Notch2 signaling is required for the development of the EpCAM^+^ DCIR2^+^ cDC2s preceding IL-23 expression

**Figure 7.**
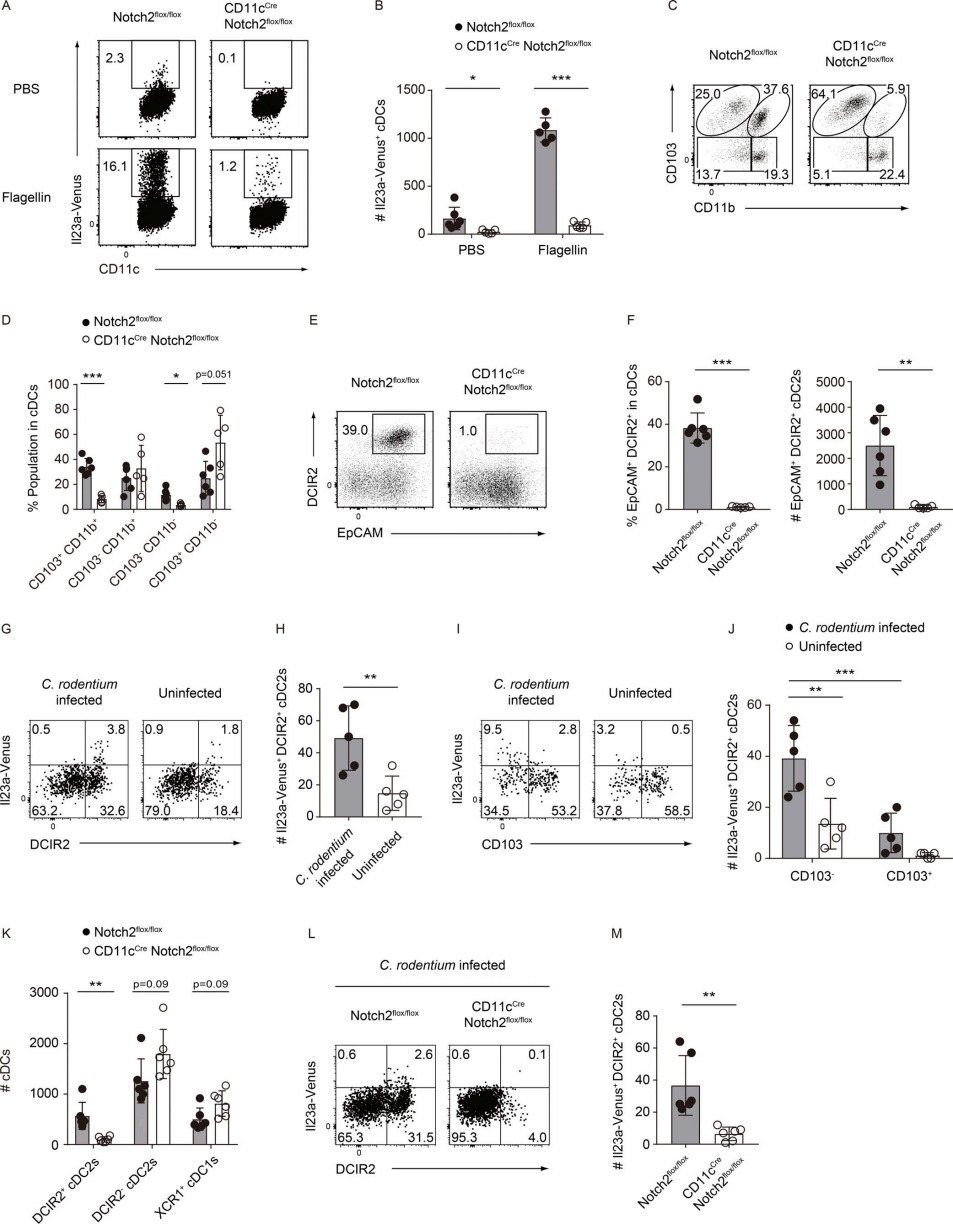
**Notch2 signaling is required for the development of the EpCAM**^**+**^ **DCIR2**^**+**^ **cDC2s preceding IL-23 expression in the gut. (A)** The frequency of Il23a-Venus expression in SILP cDCs from Notch2^flox/flox^ and CD11c^Cre^ Notch2^flox/flox^ mice 4 h after i.v. injection of PBS or flagellin. **(B)** The total cell numbers of Il23a-Venus^+^ cells in SILP cDCs (*n* = 5–6). **(C)** The frequency of four cDC subpopulations (CD103^+^ CD11b^+^, CD103^−^ CD11b^+^, CD103^−^ CD11b^−^, and CD103^+^ CD11b^−^) in SILP cDCs from Notch2^flox/flox^ and CD11c^Cre^ Notch2^flox/flox^ mice. **(D)** The percentages of the indicated cDC subpopulations from the SILP (*n* = 5–6). **(E)** The frequency of EpCAM^+^ DCIR2^+^ cells in SILP cDCs from Notch2^flox/flox^ and CD11c^Cre^ Notch2^flox/flox^ mice. **(F)** The percentages and total cell numbers of the EpCAM^+^ DCIR2^+^ cDC2s (*n* = 5–6). **(G)** The frequency of DCIR2 and Il23a-Venus in LILP cDCs 12 days after *C. rodentium* infection. **(H)** The total cell numbers of the LILP Il23a-Venus^+^ DCIR2^+^ cDC2s (*n* = 5). **(I)** The frequency of Il23a-Venus and CD103 expression in the LILP DCIR2^+^ cDC2s 12 days after *C. rodentium* infection. **(J)** The total cell numbers of the Il23a-Venus^+^ DCIR2^+^ CD103^−^ or CD103^+^ cDC2s from the LILP of uninfected and infected mice (*n* = 5). **(K)** The total cell numbers of the LILP DCIR2^+^ cDC2s, DCIR2^−^ cDC2s, and XCR1^+^ cDC1s from Notch2^flox/flox^ and CD11c^Cre^ Notch2^flox/flox^ mice 12 days after *C. rodentium* infection (*n* = 6). **(L)** The frequency of DCIR2 and Il23a-Venus expression in LILP cDCs from Notch2^flox/flox^ and CD11c^Cre^ Notch2^flox/flox^ mice 12 days after *C. rodentium* infection. **(M)** The total cell numbers of LILP Il23a-Venus^+^ DCIR2^+^ cDC2s from Notch2^flox/flox^ and CD11c^Cre^ Notch2^flox/flox^ mice 12 days after *C. rodentium* infection (*n* = 6). The data in G, I, and L are representative of two independent experiments, and the data in H, J, K, and M are pooled from two independent experiments. The data in A, C, and E are representative of three independent experiments, and the data in B, D, and F are pooled from more than three independent experiments. Statistical analyses were performed by multiple *t* test comparing Notch2^flox/flox^ and CD11c^Cre^ Notch2^flox/flox^ mice (B, D, and K), by Student’s *t* test (F, H, and M), and by two-way ANOVA followed by Tukey’s multiple comparisons test (J). *P < 0.05, **P < 0.01, ***P < 0.001. Graphs depict mean ± SD.

**Figure S4.**
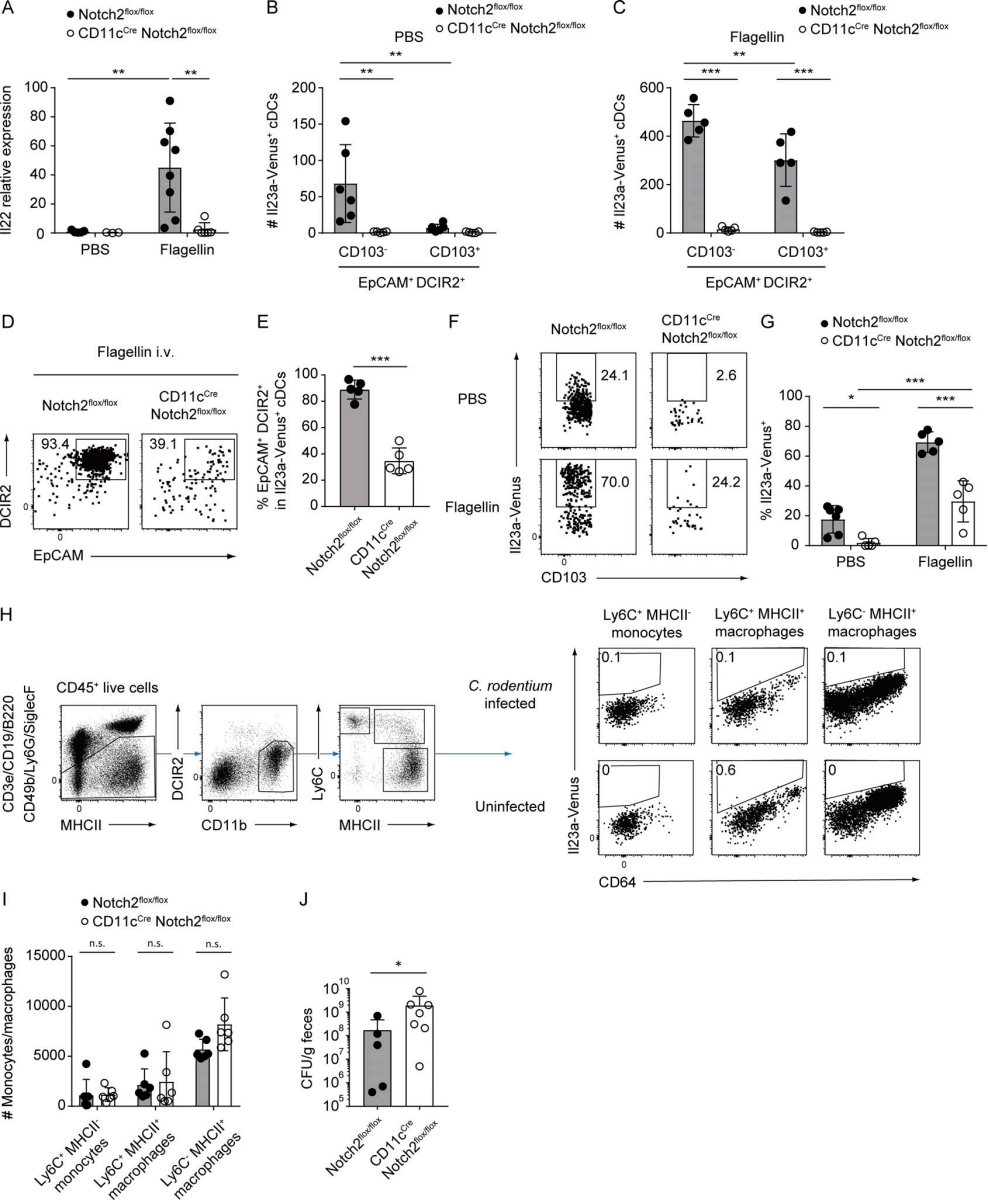
**Monocytes and macrophages do not express Il23a-Venus and are not affected in CD11c**^**Cre**^ **Notch2**^**flox/flox**^ **mice during *C. rodentium* infection. (A)** The relative expression levels of Il22 in the SILP tissues from Notch2^flox/flox^ or CD11c^Cre^ Notch2^flox/flox^ mice 2 h after i.v. injection of PBS or flagellin (*n* = 3–8). **(B and C)** The total cell numbers of Il23a-Venus^+^ EpCAM^+^ DCIR2^+^ CD103^−^ or CD103^+^ cDCs from the SILP in PBS- (B) or flagellin- (C) injected Notch2^flox/flox^ or CD11c^Cre^ Notch2^flox/flox^ mice (*n* = 5–6). **(D)** The frequency of EpCAM^+^ DCIR2^+^ cells in Il23a-Venus^+^ cDCs from the SILP of flagellin-injected Notch2^flox/flox^ or CD11c^Cre^ Notch2^flox/flox^ mice. **(E)** The percentages of EpCAM^+^ DCIR2^+^ cells in Il23a-Venus^+^ cDCs from the SILP (*n* = 5). **(F)** The frequency of Il23a-Venus^+^ cells in EpCAM^+^ DCIR2^+^ CD103^−^ CD11b^−^ cDCs from the SILP of PBS- or flagellin-injected Notch2^flox/flox^ or CD11c^Cre^ Notch2^flox/flox^ mice. **(G)** The percentages of Il23a-Venus^+^ cDCs in EpCAM^+^ DCIR2^+^ CD103^−^ CD11b^−^ cDCs from the SILP (*n* = 5–6). **(H)** The frequency of Il23a-Venus expression in different subsets of LILP monocytes and macrophages defined by the expression levels of Ly6C and MHCII. Live CD45^+^ CD19^−^ B220^−^ CD49b^−^ Ly6G^−^ SiglecF^−^ DCIR2^−^ CD11b^+^ cells were gated as monocyte/macrophage subsets, followed by separation based on the expression levels of Ly6C and MHCII. The frequency of Il23a-Venus expression was determined for three different subsets of monocytes and macrophages (Ly6C^+^ MHCII^−^ monocytes, Ly6C^+^ MHCII^+^ macrophages, and Ly6C^−^ MHCII^+^ macrophages) from LILP of *Il23a*^Venus^ mice 12 days after *C. rodentium* infection. **(I)** The total cell numbers of Ly6C^+^ MHCII^−^ monocytes, Ly6C^+^ MHCII^+^ macrophages, or Ly6C^−^ MHCII^+^ macrophages in the LILP of Notch2^flox/flox^ and CD11c^Cre^ Notch2^flox/flox^ mice 12 days after *C. rodentium* infection (*n* = 6). **(J)** CFUs of *C. rodentium* in the feces of Notch2^flox/flox^ or CD11c^Cre^ Notch2^flox/flox^ mice 12 days after infection (*n* = 5–6). Data are representative of two independent experiments in H and J and are pooled from two independent experiments in A and I. Data are representative of three independent experiments in D and F and are pooled from three independent experiments in B, C, E, and G. Statistical analyses were performed by two-way ANOVA followed by Tukey’s multiple comparisons test (A–C and G), by multiple *t* test comparing Notch2^flox/flox^ and CD11c^Cre^ Notch2^flox/flox^ mice (I), by Mann–Whitney test (J), and by Student’s *t* test (E). *P < 0.05, **P < 0.01, ***P < 0.001. n.s., not significant. Graphs depict mean ± SD.

To scrutinize the specific effects of Notch2 signaling on the development of IL-23–producing cDCs, we generated *Il23a*^Venus^ CD11c^Cre^ Notch2^flox/flox^ mice. Upon flagellin challenge, both the percentage and total cell number of Il23a-Venus^+^ cells among SILP cDCs were significantly lower in CD11c^Cre^ Notch2^flox/flox^ mice than in control Notch2^flox/flox^ mice (Fig. 7, A and B). These findings were corroborated by a marked reduction in the expression of *Il22* in SILP tissues from CD11c^Cre^ Notch2^flox/flox^ mice after flagellin injection (Fig. S4 A). Additionally, we investigated which cDC subpopulations were altered in CD11c^Cre^ Notch2^flox/flox^ mice. In line with previous reports (Satpathy et al., 2013; Lewis et al., 2011), we observed a significant reduction in the proportion of CD103^+^ CD11b^+^ cDC2s in CD11c^Cre^ Notch2^flox/flox^ mice. Furthermore, we noted that the proportion of the CD103^−^ CD11b^−^ cDC2s, a key source of IL-23 among cDC2s, was significantly reduced in CD11c^Cre^ Notch2^flox/flox^ mice (Fig. 7, C and D). Notably, the proportion and number of the EpCAM^+^ DCIR2^+^ cDC2s, encompassing both IL-23–producing CD103^+^ and CD103^−^ cells, were almost entirely diminished in CD11c^Cre^ Notch2^flox/flox^ mice (Fig. 7, E and F; and Fig. S4, B and C). It is noteworthy that there was a minor fraction of IL-23–expressing cDCs independently of Notch2 signaling, and IL-23 expression in these cells did not exhibit a strong association with the expression of EpCAM and DCIR2 (Fig. S4, D and E). The expression of Il23a-Venus in the remaining EpCAM^+^ DCIR2^+^ CD103^−^ CD11b^−^ cDC2s from CD11c^Cre^ Notch2^flox/flox^ mice was upregulated after flagellin injection (Fig. S4, F and G).

Next, we employed a *C. rodentium* infection model to investigate the kinetics of Il23a-Venus expression in gut cDC subsets. On day 12 after infection, we observed selective upregulation of Il23a-Venus within the DCIR2^+^ cDC2s in LILP tissues while DCIR2^−^ cDC2s and monocytes/macrophages did not express Il23a-Venus (Fig. 7, G and H; and Fig. S4 H). Notably, the DCIR2^+^ CD103^−^ cDC2s contained a larger number of Il23a-Venus^+^ cells than the CD103^+^ cDC2s, which was similar to the phenotypes of IL-23–producing cDC2s in the SILP at steady state and after flagellin injection (Fig. 7, I and J). Furthermore, we evaluated Il23a-Venus expression in gut cDC subsets from CD11c^Cre^ Notch2^flox/flox^ mice during *C. rodentium* infection. The DCIR2^+^ cDC2s, which included Il23a-Venus^+^ cells, were selectively affected in CD11c^Cre^ Notch2^flox/flox^ mice during *C. rodentium* infection, while cDC1s, DCIR2^−^ cDC2s, and monocytes/macrophages were unaffected (Fig. 7, K–M; and Fig. S4 I). Consistent with the loss of IL-23–producing cDCs crucial for this model, fecal *C. rodentium* titers were significantly increased in CD11c^Cre^ Notch2^flox/flox^ mice compared with control Notch2^flox/flox^ mice (Fig. S4 J). Taken together, our findings suggest that Notch2 signaling plays an essential role in the development of the EpCAM^+^ DCIR2^+^ cDC2s in the gut, which likely serves as a precursor leading to IL-23–producing CD103^+^ and CD103^−^ cDC2s. The loss of this Notch2-dependent subpopulation results in a defect in the gut IL-23–IL-22 axis and thereby increases susceptibility to *C. rodentium* infection.

### Retinoic acid signaling regulates the terminal differentiation of EpCAM^+^ DCIR2^+^ CD103^−^ cDC2s into IL-23**–**producing cDC2s in the gut

**Figure 8.**
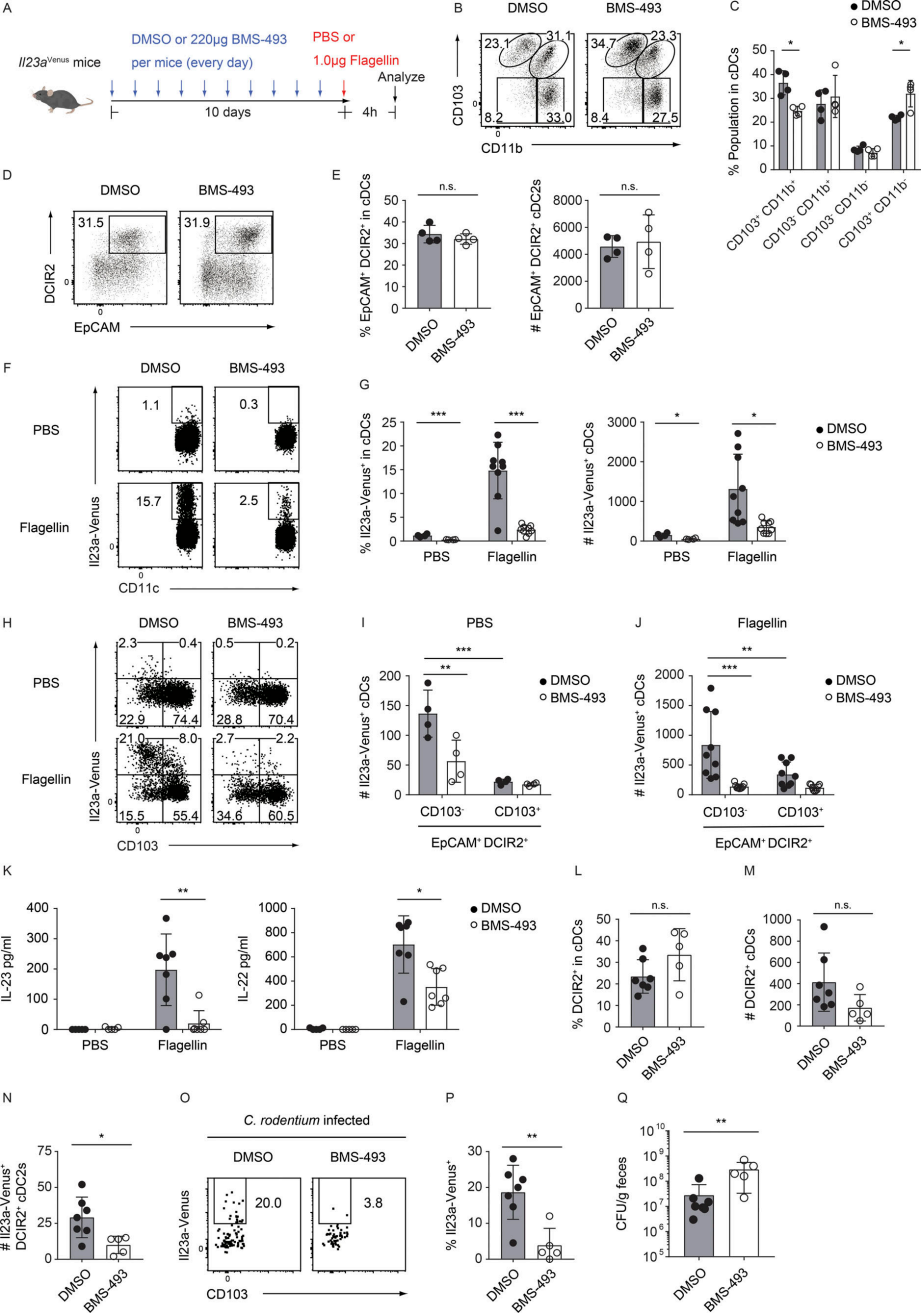
**Retinoic acid signaling controls the terminal differentiation of EpCAM**^**+**^ **DCIR2**^**+**^ **CD103**^**−**^ **cDC2s into IL-23–producing cDC2s in the gut. (A)** Experimental scheme for in vivo treatment with the pan-RARs inverse agonist (BMS-493). *Il23a*^Venus^ mice were treated with 220 μg of BMS-493 or a vehicle (DMSO) every day for 10 days. After this treatment, SILP cDC subsets and serum protein levels were analyzed 4 h after injection of PBS or 1 μg of flagellin. **(B)** The frequency of four cDC subpopulations (CD103^+^ CD11b^+^, CD103^−^ CD11b^+^, CD103^−^ CD11b^−^, and CD103^+^ CD11b^−^) in SILP cDCs from mice treated with either BMS-493 or vehicle. **(C)** The percentages of the indicated cDC subsets in the SILP (*n* = 4). **(D)** The frequency of EpCAM^+^ DCIR2^+^ cells in SILP cDCs from mice treated with either BMS-493 or vehicle. **(E)** The percentages and total cell numbers of the EpCAM^+^ DCIR2^+^ cDC2s (*n* = 4). **(F)** The frequency of Il23a-Venus expression in SILP cDCs after injection of PBS or flagellin in mice treated with either BMS-493 or vehicle. **(G)** The percentages and total cell numbers of Il23a-Venus^+^ cells in SILP cDCs (*n* = 4–9). **(H)** The frequency of Il23a-Venus and CD103 expression in the SILP EpCAM^+^ DCIR2^+^ cDC2s from mice treated with either BMS-493 or vehicle after PBS or flagellin injection. **(I and J)** The total cell numbers of the Il23a-Venus^+^ EpCAM^+^ DCIR2^+^ CD103^−^ or CD103^+^ cDC2s from the SILP in PBS- (I) or flagellin- (J) injected mice (*n* = 4–9). **(K)** The serum protein levels of IL-23 and IL-22 in mice treated with either BMS-493 or vehicle after PBS or flagellin injection (*n* = 4–7). **(L)** The percentages of DCIR2^+^ cells in LILP cDCs from mice treated with either BMS-493 or vehicle 12 days after *C. rodentium* infection (*n* = 5–7). **(M)** The total cell numbers of the LILP DCIR2^+^ cDC2s from mice treated with either BMS-493 or vehicle 12 days after *C. rodentium* infection (*n* = 5–7). **(N)** The total cell numbers of the Il23a-Venus^+^ DCIR2^+^ cDC2s from mice treated with either BMS-493 or vehicle 12 days after *C. rodentium* infection (*n* = 5–7). **(O)** The frequency of Il23a-Venus expression in the LILP DCIR2^+^ CD103^−^ CD11b^−^ cDC2s from mice treated with either BMS-493 or vehicle 12 days after *C. rodentium* infection. **(P)** The percentages of Il23a-Venus^+^ cells in the LILP DCIR2^+^ CD103^−^ CD11b^−^ cDC2s 12 days after *C. rodentium* infection (*n* = 5–7). **(Q)** CFUs of *C. rodentium* in the feces of mice treated with either BMS-493 or vehicle 12 days after *C. rodentium* infection (*n* = 5–7). The data in O are representative of two independent experiments, and the data in K–N, P, and Q are pooled from two independent experiments. The data in B, D, F, and H are representative of three independent experiments, and the data in C, E, G, I, and J are pooled from three independent experiments. Statistical analyses were performed by multiple *t* test comparing DMSO- and BMS-493–treated mice (C, G, and K), by Student’s *t* test (E, L, M, N, and P), by Mann–Whitney test (Q), and by two-way ANOVA followed by Tukey’s multiple comparisons test (I and J). *P < 0.05, **P < 0.01, ***P < 0.001. n.s., not significant. Graphs depict mean ± SD.

To verify the impact of retinoic acid signaling on the differentiation of IL-23–producing gut cDCs that display a gene profile indicative of retinoic acid signaling, we administered BMS-493, a pan-retinoic acid receptor (RAR) inverse agonist (Germain et al., 2009), to *Il23a*^Venus^ mice daily for 10 days (Fig. 8 A). As expected, on the basis of previous reports (Klebanoff et al., 2013; Zeng et al., 2016), treatment with BMS-493 resulted in a reduction in the proportion of the CD103^+^ CD11b^+^ cDC2s among cDCs (Fig. 8, B and C), while the percentage and total number of the EpCAM^+^ DCIR2^+^ cDC2s, which is regulated by Notch2 signaling, remained comparable between the control and BMS-493–treated groups (Fig. 8, D and E). We subsequently evaluated Il23a-Venus expression in gut cDCs with or without BMS-493 treatment at steady state or after flagellin stimulation. In both cases, the inhibition of retinoic acid signaling led to a significant decrease in the percentage and total number of Il23a-Venus^+^ cDCs (Fig. 8, F and G). Notably, the number of Il23a-Venus^+^ EpCAM^+^ DCIR2^+^ CD103^−^ cDC2s was also significantly decreased in BMS-493–treated mice under both steady state and flagellin-challenged conditions (Fig. 8, H–J). Moreover, treatment with BMS-493 resulted in a marked disruption of the IL-23–IL-22 axis, which was driven by IL-23 production by gut cDCs following flagellin injection (Fig. 8 K).

We next administered BMS-493 to *Il23a*^Venus^ mice that had been infected with *C. rodentium*. This treatment did not significantly affect the percentage and number of DCIR2^+^ cDC2s in LILP from *C. rodentium*–infected mice. However, it resulted in a significant decrease in the number of Il23a-Venus^+^ DCIR2^+^ cDCs (Fig. 8, L–N). Furthermore, the Il23a-Venus expression in DCIR2^+^ CD103^−^ CD11b^−^ cDC2s was significantly decreased in BMS-493–treated mice, similar to the observation in SILP cDCs at steady state and after flagellin challenge (Fig. 8, O and P). Consistent with the loss of IL-23 expression in Notch2-dependent DCIR2^+^ cDCs crucial for this model, fecal *C. rodentium* titers were significantly increased in BMS-493–treated mice (Fig. 8 Q).

**Figure S5.**
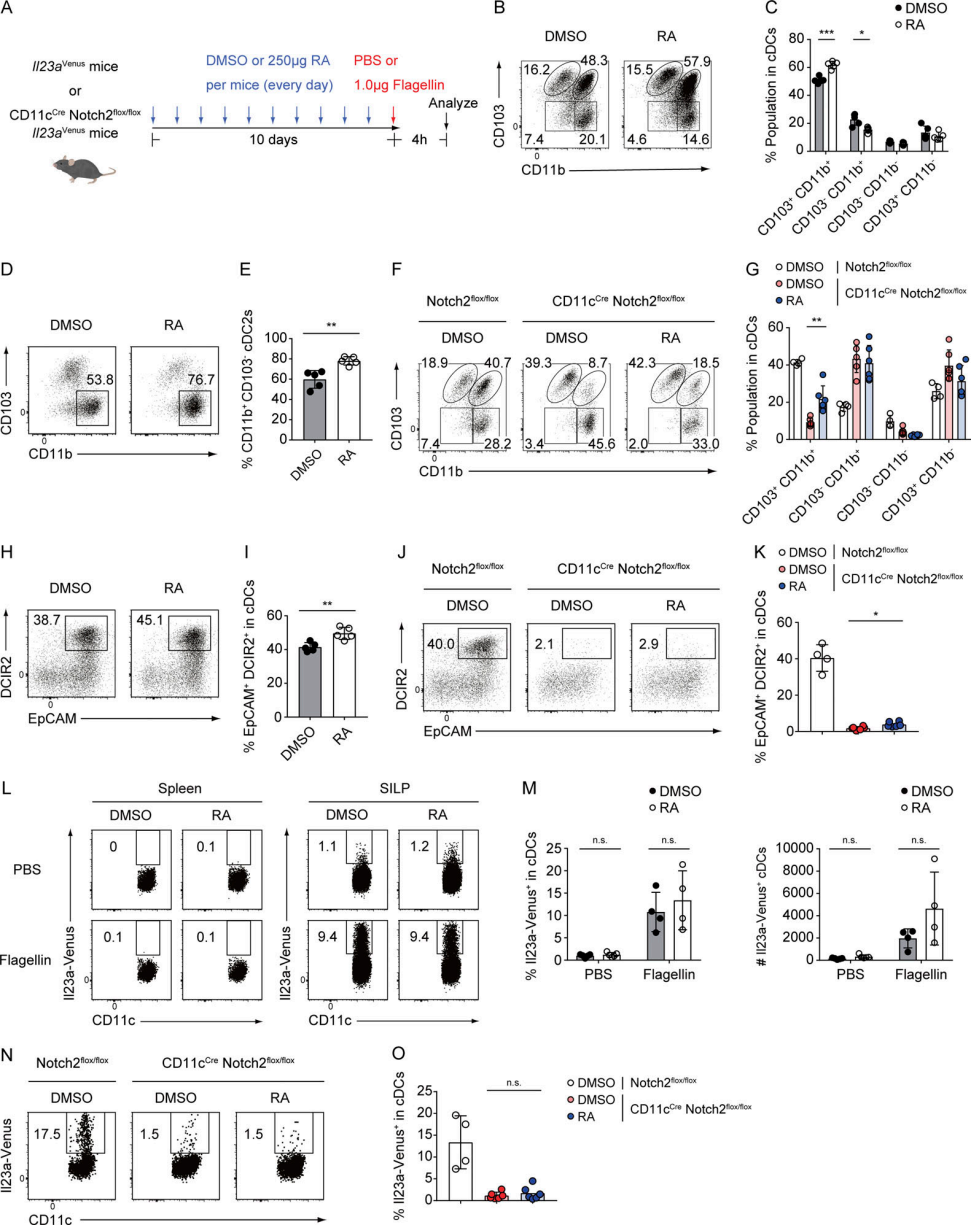
**Retinoic acid supplementation has no effect on Il23a-Venus expression. (A)** Experimental scheme for in vivo supplementation of retinoic acid (RA). *Il23a*^Venus^ mice or CD11c^Cre^ Notch2^flox/flox^ *Il23a*^Venus^ mice were treated with 250 μg of RA or a vehicle (DMSO) every day for 10 days. After this treatment, splenic and SILP cDC subsets were analyzed 4 h after injection of PBS or 1 μg of flagellin. **(B)** The frequency of four cDC subpopulations (CD103^+^ CD11b^+^, CD103^−^ CD11b^+^, CD103^−^ CD11b^−^, and CD103^+^ CD11b^−^) in the SILP cDCs from *Il23a*^Venus^ mice treated with either RA or vehicle. **(C)** The percentages of the indicated cDC subsets in the SILP from *Il23a*^Venus^ mice (*n* = 5). **(D)** The frequency of CD11b^+^ CD103^−^ cDC2s in splenic cDCs from *Il23a*^Venus^ mice treated with either RA or vehicle. **(E)** The percentages of CD11b^+^ CD103^−^ cDC2s in splenic cDCs from *Il23a*^Venus^ mice (*n* = 5). **(F)** The frequency of the indicated four cDC subpopulations in SILP cDCs from Notch^flox/flox^ *Il23a*^Venus^ mice treated with vehicle and CD11c^Cre^ Notch^flox/flox^ *Il23a*^Venus^ mice treated with either RA or vehicle. **(G)** The percentages of the indicated cDC subsets in SILP cDCs from Notch^flox/flox^ *Il23a*^Venus^ mice or CD11c^Cre^ Notch^flox/flox^ *Il23a*^Venus^ mice (*n* = 4–6). **(H)** The frequency of EpCAM^+^ DCIR2^+^ cDCs in SILP cDCs from *Il23a*^Venus^ mice treated with either RA or vehicle. **(I)** The percentages of EpCAM^+^ DCIR2^+^ cDCs in SILP cDCs from *Il23a*^Venus^ mice (*n* = 5). **(J)** The frequency of EpCAM^+^ DCIR2^+^ cDCs in SILP cDCs from Notch^flox/flox^ *Il23a*^Venus^ mice treated with vehicle and CD11c^Cre^ Notch^flox/flox^ *Il23a*^Venus^ mice treated with either RA or vehicle. **(K)** The percentages of EpCAM^+^ DCIR2^+^ cDCs in SILP cDCs from Notch^flox/flox^ *Il23a*^Venus^ mice or CD11c^Cre^ Notch^flox/flox^ *Il23a*^Venus^ mice (*n* = 4–6). **(L)** The frequency of Il23a-Venus expression in splenic and SILP cDCs after injection of PBS or flagellin in *Il23a*^Venus^ mice treated with either RA or vehicle. **(M)** The percentages and total cell numbers of Il23a-Venus^+^ cells in SILP cDCs from *Il23a*^Venus^ mice (*n* = 4–5). **(N)** The frequency of Il23a-Venus expression in SILP cDCs after injection of flagellin in Notch^flox/flox^ *Il23a*^Venus^ mice treated with vehicle and CD11c^Cre^ Notch^flox/flox^ *Il23a*^Venus^ mice treated with either RA or vehicle. **(O)** The percentages of Il23a-Venus^+^ cells in SILP cDCs after injection of flagellin (*n* = 4–6). The data in B, D, F, H, J, L, and N are representative of two independent experiments, and the data in C, E, G, I, K, M, and O is pooled from two independent experiments. Statistical analyses were performed by multiple *t* test comparing DMSO- and RA-treated mice (C, G, and M), and by Student’s *t* test comparing DMSO- and RA-treated mice (E, I, K, and O). *P < 0.05, **P < 0.01, ***P < 0.001. n.s., not significant. Graphs depict mean ± SD.

We also investigated whether retinoic acid supplementation affects IL-23 expression in cDC2s from the SILP and spleen. We administered retinoic acid to *Il23a*^Venus^ or *Il23a*^Venus^ CD11c^Cre^ Notch2^flox/flox^ mice daily for 10 days (Fig. S5 A). Retinoic acid supplementation significantly increased the percentages of CD103^+^ CD11b^+^ cDC2s in SILP and CD11b^+^ CD103^−^ cDC2s in the spleen, consistent with the previous reports (Klebanoff et al., 2013; Zeng et al., 2016; Fig. S5, B–G). Additionally, there was a notable increase in the percentages of EpCAM^+^ DCIR2^+^ cDC2s in SILP from both retinoic acid–treated WT mice and CD11c^Cre^ Notch2^flox/flox^ mice (Fig. S5, H–K). Conversely, the percentages and numbers of Il23a-Venus^+^ cDCs were not significantly altered in both splenic and SILP cDCs of WT or CD11c^Cre^ Notch2^flox/flox^ mice (Fig. S5, L–O). These data indicate that the basal levels of retinoic acid at steady state appeared to be sufficient for inducing IL-23 expression by EpCAM^+^ DCIR2^+^ SILP cDCs. These findings support a critical role for homeostatic retinoic acid signaling in the terminal differentiation of IL-23–producing gut cDC2s, presumably originating from the Notch2-dependent EpCAM^+^ DCIR2^+^ cDC2s.

## Discussion

This study presents compelling evidence regarding the developmental and functional heterogeneity of the cDC2 subset in maintaining gut homeostasis under pathological conditions. We also identified the molecular basis of the IL-23–producing cDC2s in the gut; Notch2 signaling is critical for the development of DCIR2^+^ EpCAM^+^ cDC2s, which contain the bulk of IL-23–producing cDCs. Furthermore, additional stimulation with retinoic acid is necessary for the acquisition of IL-23 production capability, and IL-23–producing cDC2s are confined to GALTs. While the terminally differentiated cDC2 subpopulation physiologically senses microbial products via TLR5 and secretes IL-23, thereby maintaining gut homeostasis, the invasion of pathogenic attaching and effacing bacteria triggers the activation of IL-23–producing cDC2s, driving robust activation of the IL-23–IL-22 axis as a host defense mechanism. Taken together, our findings provide crucial insights into the developmental pathways and cellular dynamics of IL-23–producing cDC2s under steady-state conditions and in response to pathogen infection.

Despite the importance of IL-23 under physiological and pathological conditions, the cellular source of IL-23 in MNPs has remained controversial and incompletely defined for many years (Eken and Oukka, 2016; Luciani et al., 2022). Previous studies have attempted to assess the importance of macrophages and cDCs in the production of IL-23 and host defense against *C. rodentium* infection using various mouse models. However, these attempts have not provided a definitive answer to date due to conflicting interpretations. Previous reports showed cDCs, rather than macrophages, play a pivotal role as the cellular source of IL-23 in the gut to regulate *C. rodentium* infection using *Zbtb46*^DTR^ and *Flt3l*^−/−^ mice, which specifically target cDCs (Kinnebrew et al., 2012; Satpathy et al., 2013). Conversely, in separate papers, it was reported that monocyte-derived Cx3cr1^+^ MNPs serve as the primary source of IL-23, in contrast to cDCs, using Cx3cr1^DTR^ mice. Another study suggested that both Cx3cr1-expressing macrophages and cDCs are involved in IL-23 production in the host defense against *C. rodentium* infection (Longman et al., 2014; Aychek et al., 2015). The variation in the severity of *C. rodentium* infection between previous studies and our results might be attributed, at least in part, to differences in the environment of specific pathogen–free animal facilities, particularly gut microbiota. It is well-established that gut microbiota can influence the kinetics of *C. rodentium* colonization, thereby impacting the overall disease severity (Osbelt et al., 2020). In this study, we utilized *Il23a*^Venus^ mice to enable direct visualization of IL-23–expressing cells at the single-cell level. Our results indicated that monocytes and macrophages did not express Il23a-Venus under steady-state conditions or after *C. rodentium* challenge. Furthermore, the selective depletion of gut DCIR2^+^ cDCs in vitro supported the notion that IL-23 from cDCs, but not macrophages, was critical for the induction of IL-22. We also provided evidence that these IL-23–producing gut DCIR2^+^ cells were bona fide cDCs by tracing their development from pre-cDCs, and assessing the expression of specific cDC markers that distinguish them from macrophages. Based on our findings, we conclude that among MNPs, gut cDC2s are the crucial cellular source of IL-23 during *C. rodentium* infection.

The identification of the gut cDC2 subpopulation responsible for IL-23 production in mucosal host defense has been challenging due to the heterogeneous properties of cDC2s. A previous study using CD11c^Cre^ Notch2^flox/flox^ mice showed that CD103^+^ CD11b^+^ cDC2s, whose development relies on Notch2 signaling, are the crucial source of IL-23 for host protection against *C. rodentium* infection (Kinnebrew et al., 2012; Satpathy et al., 2013). However, a different mouse model revealed that compared with WT mice, human Langerin promoter-DTA transgenic mice (hLangerin^DTA^ mice), which also lack CD103^+^ CD11b^+^ cDC2s, showed comparable resistance to *C. rodentium* infection and potent *Il22* expression in SILP tissues following flagellin challenge (Welty et al., 2013). These findings imply that a small population of Notch2-dependent cDC2s, which was not previously identified in CD11c^Cre^ Notch2^flox/flox^ mice, could constitute the primary source of IL-23 production. Our reporter system revealed that in addition to CD103^+^ CD11b^+^ cDC2s, the EpCAM^+^ DCIR2^+^ CD103^−^ CD11b^−^ cDC2s predominantly expressed Il23a-Venus in *C. rodentium*– or flagellin-challenged mice. Further analysis of *Il23a*^Venus^ CD11c^Cre^ Notch2^flox/flox^ mice demonstrated that IL-23–expressing cDC2s, defined by not only CD103^+^ CD11b^+^ cDCs but also EpCAM^+^ DCIR2^+^ CD103^−^ CD11b^−^ cDCs, are affected by a defect in Notch2 signaling. In contrast, it is likely that the IL-23–expressing EpCAM^+^ DCIR2^+^ CD103^−^ CD11b^−^ cDC2s remain unaffected in hLangerin^DTA^ mice based on specific control of DTA expression by the human langerin promoter in the CD103^+^ CD11b^+^ cDC2 subpopulation. Additionally, EpCAM^+^ DCIR2^+^ CD11b^−^ CD103^−^ and CD11b^+^ CD103^+^ IL-23–expressing cDC2s appear to develop independently from their precursors, and the phenotypic transition between CD11b^−^ CD103^−^ and CD11b^+^ CD103^+^ IL-23–expressing cDC2s appears to be a limited phenomenon (Welty et al., 2013). Furthermore, our findings, which identify EpCAM and DCIR2 as reliable markers for IL-23–expressing cDC2s, reconcile previous contradictory findings and deepen our understanding of the functional heterogeneity of cDC2s with IL-23–producing properties.

Diverse subpopulations of gut cDCs are strategically positioned at discrete sites to regulate specific immune responses. The majority of CD103^+^ CD11b^+^ cDC2s are found within lamina propria tissues of villi or crypts, where they regulate adaptive immunity by inducing effector Th17 or Foxp3^+^ regulatory T cells (Schlitzer et al., 2013; Welty et al., 2013; Luciani et al., 2022). In contrast, the LyzM^+^ cDC2 subpopulation can mediate a noncanonical function, modulating lipid metabolism by promoting free fatty acid transporter expression by enterocytes (Guendel et al., 2020). The exact localization of IL-23–producing cDCs, however, has not been well defined. By employing whole-mount staining analysis of the small and large intestines from *Il23a*^Venus^ mice, our results clearly demonstrate that IL-23–producing cDCs are exclusively located in GALTs, such as cryptopatches and ILFs, but not in lamina propria tissues of villi or crypts. These specialized IL-23–producing cDC2s, which are colocalized with IL-22–producing ILC3s (Ebertl and Litman, 2004), could serve as frontline sentinels to monitor commensal and enteropathogenic bacteria.

In this study, we propose a two-step model of development for IL-23–producing cDC2s, which are critical for maintaining gut immune homeostasis. Our findings indicate that combined Notch2 and retinoic acid receptor signaling pathways, although the specific order remains to be fully elucidated, are essential for the development of the EpCAM^+^ DCIR2^+^ cDC2s and their terminal differentiation with a potent capacity for IL-23 production. Retinoic acid, a metabolite of vitamin A, is crucial for controlling innate and adaptive immune cells in the gut (Mora et al., 2008; Beijer et al., 2014; Wilhelm et al., 2017). Vitamin A deficiency is prevalent in developing countries and a major public health problem that results in a high prevalence and mortality rate of intestinal infectious diseases caused by bacteria such as *Salmonella* spp., *Shigella* spp., and enteropathogenic *E. coli* in young children (World Health Organization, 2009; Villamor et al., 2000; Hossain et al., 1998). Moreover, previous studies have shown that IL-22–producing ILC3s are impaired in vitamin A–deficient mice and after BMS-493 treatment, leading to increased susceptibility to *C. rodentium* infection (Spencer et al., 2014; McDaniel et al., 2015). It is beyond the scope of the current study to determine whether retinoic acid has cell-intrinsic or -extrinsic effects on the cDC2 subset, but our gene expression analysis indicates that IL-23–producing cDCs express components, including retinol receptors, alcohol dehydrogenases, and CRABPII, necessary for metabolizing retinol into retinoic acid and integrating its signaling pathway (Kam et al., 2012; Napoli, 2016). Conversely, in addition to that secreted by intestinal epithelial cells and the cDC1s (Grizotte-Lake et al., 2018; Coombes et al., 2007; Luda et al., 2016), retinoic acid secreted by IL-23–producing cDC2s may also contribute to the maintenance of ILC3s within GALTs. Our research findings provide novel insights into the previously unrecognized role of retinoic acid in the development of IL-23–producing cDCs, which is the key upstream regulator of IL-22 production by ILC3s.

Given the implication of the inflammatory cytokine IL-23 in the pathogenesis of autoimmune and inflammatory diseases, such as inflammatory bowel disease, psoriasis, psoriatic arthritis, and multiple sclerosis (Teng et al., 2015), it is imperative to investigate how the development and function of IL-23–producing cDCs can be modulated under pathogenic conditions. To this end, the *Il23a*^Venus^ mouse model would be a valuable tool for assessing the augmentation or attenuation of IL-23 production by cDCs. However, the identity and functional regulation of pathogenic IL-23–producing cDCs, similar to their physiological counterparts in the gut, remain largely unknown. Therefore, future studies are needed to elucidate the differences in differentiation and functional regulation between IL-23–producing cDCs under physiological and pathogenic conditions. The identification of specific regulators or targeted compounds in the future may facilitate the development of a tailored therapeutic strategy for the treatment of infectious and autoimmune diseases.

## Materials and methods

### Mice

WT C57BL/6J mice were purchased from CLEA Japan. *Il23a*^Venus^ mice and *Il23a*^−/−^ mice lacking the exon 1–4 region of *Il23a* were generated using the CRISPR/Cas9 system. The microinjection of one crRNA (FASMAC) and IRES2-Venus-SV40late polyA sequence with homology arms for *Il23a*^Venus^ mice, or two crRNAs for *Il23a*^−/−^ mice, along with tracrRNA (FASMAC) and purified recombinant Cas9 (Thermo Fisher Scientific), was carried out in in vitro fertilized C57BL/6 mouse eggs. CD11c^Cre^ mice (B6.Cg-Tg(Itgax-cre)1-1Reiz/J) were purchased from the Jackson Laboratory (Caton et al., 2007). Notch2^flox/flox^ mice (B6;Cg-Notch2<tm1.1Hhi>, RBRC09647) were obtained from the RIKEN Bioresource Center (Saito et al., 2003). All mice were on a C57BL/6 background and were maintained under specific pathogen–free conditions. Both male and female mice at 6–12 wk of age were used for all the experiments. All animal experiments were approved by the Ethical Committee of the Institute for Life and Medical Sciences, Kyoto University, and all relevant experiments were performed in accordance with institutional guidelines.

### Preparation of single-cell suspensions from various tissues

To obtain single-cell suspensions from the lamina propria of intestines, the Peyer’s patches from the small intestine were removed. Subsequently, the intestinal tissues were opened longitudinally and cut into 1-cm pieces, followed by washing in 50 ml of PBS. To remove the epithelial layers, the tissues were incubated on a magnetic shaker (Fine DC stirrer G-1; Tokyo Glass Kikai) in RPMI medium (Sigma-Aldrich) containing 2% FBS, 5 mM EDTA (Nacalai Tesque), and 1 mM DL-dithiothreitol (Wako) at 37°C for 15 min. The tissues were then agitated with a magnetic stir bar in a 50-ml centrifuge tube containing 20 ml of 2% FBS/RPMI. The remaining intestinal tissues were digested using a magnetic shaker in 12 ml of RPMI medium containing 2% FBS, 0.1 mg/ml DNase I (Roche), and 0.5 mg/ml collagenase (Wako) at 37°C for 1 h. The tissues were homogenized and the resulting mixture was filtered through a 70-μm cell strainer.

To prepare single-cell suspensions from the spleens, lungs, kidneys, livers, LNs, and Peyer’s patches, the tissues were minced with scissors and subjected to digestion using a magnetic shaker or MACSmix tube rotator (Miltenyi Biotec) in RPMI medium containing 2% FBS, 0.1 mg/ml DNase I, and 0.5 mg/ml collagenase at 37°C for 1 h. The tissues were then passed through a 70-μm cell strainer. Immune cells in kidneys and livers were enriched using Percoll Plus (Cytiva). Finally, red blood cells were lysed using red blood cell lysis buffer (Sigma-Aldrich).

To prepare single-cell suspensions from the dermis, mouse ear skin was incubated with 5 mg/ml Dispase II (Roche) in HBSS medium (Nacalai Tesque) for 1 h. Subsequently, the epidermis and dermis were separated, and the dermis was treated with 0.1 mg/ml DNase I and 0.5 mg/ml collagenase at 37°C for 1 h. Single-cell suspensions used for flow cytometry analysis were obtained by passing the digested tissues through a 70-μm cell strainer.

### Flagellin injection

1 μg of ultrapure flagellin (FLA-ST; InvivoGen) or vehicle (PBS) was intravenously (i.v.) injected via the tail vein. For flow cytometry analysis of Il23a-Venus expression, the tissues were harvested 4 h after flagellin injection.

### In vivo BMS-493 and retinoic acid supplementation

220 μg of BMS-493 (Tocris) dissolved in 50 μl of dimethyl sulfoxide (DMSO; Sigma-Aldrich) was administered intraperitoneally to *Il23a*^Venus^ mice daily for 10 days. Control mice received a daily injection of 50 μl of DMSO for the same duration. On day 10, the mice were i.v. injected with 1 μg of flagellin or PBS and analyzed 4 h after injection. BMS-493 or DMSO was administered intraperitoneally to *Il23a*^Venus^ mice daily for 10 days, beginning on day 2 after *C. rodentium* infection. On day 12 after infection, cDCs in LILP were analyzed and colony-forming units (CFUs) of feces were calculated. 250 μg of retinoic acid (Sigma-Aldrich) dissolved in 50 μl of DMSO was administered intraperitoneally to *Il23a*^Venus^ mice or CD11c^Cre^ Notch2^flox/flox^ *Il23a*^Venus^ mice daily for 10 days.

### Quantitative RT-PCR of lamina propria tissues

A 1-cm section of small intestine tissue was collected 2 h after i.v. injection of either PBS or flagellin. The epithelial layers were removed using the method described above. The remaining lamina propria tissues were homogenized in buffer RLT (Qiagen) containing 2-mercaptoethanol (Wako) using MagNA Lyzer and MagNA Lyzer green beads (Roche). Total RNA was extracted using the RNeasy mini kit (Qiagen). Subsequently, 500 ng of total RNA was reverse transcribed into cDNA using Superscript VILO master mix (Thermo Fisher Scientific). The resultant cDNA was diluted 10-fold, and 5 μl of cDNA was used for quantitative PCR. The expression levels of *Hprt* and *Il22* were quantified using TaqMan probes (*Hprt*; Mm01545399_m1, *Il22*; Mm00444241_m1) and THUNDERBIRD Probe qPCR Mix (TOYOBO) on a StepOnePlus Real-Time PCR System (Thermo Fisher Scientific). The expression levels of *Il22* were quantified after normalization to *Hprt*.

### Flow cytometry

Single-cell suspensions were incubated with anti-mouse CD16/32 (93; BioLegend), followed by incubation with Fixable Viability Dye eFlour 506 or Fixable Viability Dye eFlour 780 (Thermo Fisher Scientific) and subsequent staining with the following fluorescent dye–conjugated monoclonal antibodies against various surface proteins: anti-mouse XCR1 (ZET; BioLegend), anti-mouse EpCAM (G8.8; BioLegend), anti-mouse ESAM (1G8/ESAM; BioLegend), anti-mouse CD8a (53-6.7; BioLegend), anti-mouse CD11c (N418; BioLegend), anti-mouse CD26 (H194-112; BioLegend), anti-mouse CD86 (GL-1; BioLegend), anti-mouse CD88 (20/70; BioLegend), anti-mouse DCIR2 (33D1; BioLegend), anti-mouse CD90.2 (30-H12; BioLegend), anti-mouse CD172a (P84; BioLegend), anti-mouse IA/IE (M5/114.15.2; BioLegend), anti-mouse CD11b (M1/70; BioLegend), anti-CD45 (30-F11; BioLegend), anti-mouse CD3e (145-2C11; BioLegend), anti-mouse B220 (RA3-6B2; BioLegend), anti-mouse CD19 (6D5; BioLegend), anti-mouse CD49b (DX5; BioLegend), anti-mouse Ly6G (1A8; BioLegend), anti-mouse SiglecF (S17007L; BioLegend), anti-mouse TLR5 (ACT5; BioLegend), anti-mouse CD103 (M290; BD Biosciences), anti-mouse F4/80 (T45-2342; BD Biosciences), anti-mouse CD64 (X54-5/7.1; BD Biosciences), anti-mouse Flt3 (A2F10.1; BD Biosciences), anti-mouse CCR2 (Y15-488.rMAb; BD Biosciences), and anti-mouse Ly6C (AL-21; BD Biosciences). Intracellular Zbtb46 was stained with anti-mouse Zbtb46 antibody (U4-1374; BD Biosciences) or the corresponding isotype control antibody (RTK2071; BioLegend) using the Foxp3/Transcription Factor Staining Buffer Set (eBioscience).

A FACSCantoII (BD Biosciences) or Cytoflex S (Beckman Coulter) was used for flow cytometry analysis and a FACSAriaⅡ (BD Biosciences) was used for cell sorting. The resulting fcs files were analyzed using FlowJo software (Tree Star, Inc.).

### Screening of surface markers highly correlated with Il23a-Venus^+^ cDC2s

Single-cell suspensions were obtained from the mLNs of *Il23a*^Venus^ mice, and CD11c^+^ cells were enriched with the MACS system. The cells were incubated with anti-CD16/32 for 15 min followed by an anti-CD11c PE-cy7 antibody for 20 min. Subsequently, anti-PE microbeads (Miltenyi Biotec) were added for 20 min and the positively selected fraction was obtained by passing the cells through the LS column (Miltenyi Biotec). The selected cells were then incubated for 20 min with Fixable Viability Dye eFluor 506 and selected markers to gate the cDC2 subset, such as anti-XCR1, anti-B220, anti-CD19, anti-CD11c, anti-CD103, anti-CD172a, anti-MHCII, anti-CD11b, anti-F4/80, and anti-CD8a, without the use of PE-conjugated antibodies. After washing, the cells were aliquoted across ∼260 wells containing PE-conjugated antibodies against distinct cell surface proteins (LEGENDScreen Mouse PE Kit; BioLegend) and incubated for 30 min. The correlation between each surface protein and Il23a-Venus expression in cDC2 subsets was analyzed by flow cytometry.

### Generation and stimulation of bone marrow**–**derived dendritic cells

Bone marrow cells were collected from the tibia and femur of *Il23a*^Venus^ mice and subsequently cultured in RPMI medium containing 10% FBS, Glutamax (Gibco), and 20 ng/ml mouse GM-CSF (PeproTech) for 6 days. The culture medium was changed every 2 days. On day 6, the differentiated dendritic cells were stimulated with 1 μg/ml LPS-EB (InvivoGen) for 24 h and analyzed by flow cytometry.

### Ce3D clarification procedures for imaging of intestinal tissues

The 1–2-cm sections of intestinal tissues were fixed with 4% paraformaldehyde (Nacalai Tesque) at 4°C for 16 h and rinsed with PBS three times. The tissue samples were incubated in 500 μl of Ce3D Permeabilization/Blocking Buffer (BioLegend) for 24 h. For the first antibody staining, the samples were incubated for 2 days with a mixture of anti-mouse/human B220 Alexa Fluor 594 (RA3-6B2; BioLegend), anti-mouse CD11c Alexa Fluor 647 (N418; BioLegend), and purified rabbit anti-GFP polyclonal antibody (598; MBL Life Science) dissolved in 500 μl of Ce3D Antibody Diluent Buffer (BioLegend), followed by three washes with 500 μl of Ce3D Wash Buffer (BioLegend). The samples were then treated with goat anti-rabbit Alexa Fluor 488 (Thermo Fisher Scientific) for 2 days, followed by three washes. During the second wash step, DAPI (Dojindo) was added to the wash buffer. Finally, the samples were incubated in Ce3D Tissue Clearing Solution (BioLegend) for 12 h. After clearing the tissue, the samples were mounted onto a sample chamber with vacuum grease used as a spacer and the space was filled with tissue-clearing solution and covered with a coverslip.

The 3D images presented in Fig. 5, C and D, were obtained using a Dragonfly 502 (Andor) equipped with a 10×/0.45 NA CFI Plan Apochromat Lambda dry objective (Nikon) and were analyzed using Imaris viewer (Oxford Instruments). The 2D images shown in Fig. 5 E were acquired using a TCS SP8 (Leica) equipped with a 40×/1.30 Oil HC Plan Apochromat CS2 objective (Nikon) and were analyzed using LAS X (Leica).

### Detection of cytokines by ELISA

Lamina propria cells were isolated from the small intestine of WT C57BL/6 or *Il23a*^−/−^ mice and immune cells were enriched using Percoll Plus (Cytiva). To prepare the DCIR2^+^ cell-depleted fractions shown in Fig. 3 I, cells were treated with a biotinylated anti-DCIR2 antibody (BioLegend) or a biotinylated isotype control antibody (RTK4530; BioLegend) for 20 min. EDTA-free buffer was used for all cell selection procedures to ensure that calcium ions were present, a requirement for anti-DCIR2 antibody binding to DCIR2. To prepare the eosinophil-depleted fractions shown in Fig. S2 H, cells were treated with a biotinylated anti-SiglecF antibody (BioLegend) or a biotinylated isotype control antibody (RTK2758; BioLegend) for 20 min. Then, the cells were incubated with anti-biotin microbeads (Miltenyi Biotec) for 20 min and passed through the LS column (Miltenyi Biotec) to obtain the negatively selected fraction. For the analysis of IL-22 production, 2.0 × 10^5^ cells were stimulated with 1 μg/ml ultrapure FLA-ST or 25 ng/ml recombinant mouse IL-23 (Thermo Fisher Scientific) for 16 h. The cell supernatant was collected and IL-22 protein was measured with ELISA MAX Deluxe Set Mouse IL-22 (BioLegend). For the analysis of IL-22 and IL-23 in serum, serum samples were collected 4 h after flagellin or PBS injection, and cytokines were measured using ELISA MAX Deluxe Set Mouse IL-22 (BioLegend) and ELISA MAX Deluxe Set Mouse IL-23 (BioLegend).

### Adoptive transfer of pre-cDCs

The adoptive transfer of pre-cDCs was performed as previously described (Bosteels et al., 2020). In brief, CD45.2^+^ *Il23a*^Venus^ mice were intraperitoneally injected every other day with 10 μg of Flt3l-Fc (BioXCell) for 8 days to expand pre-cDCs. The bone marrow was collected from the tibia, femur, pelvis, and humerus of these Flt3l-Fc–injected mice, and bone marrow cells were subsequently stained with a cocktail of biotinylated antibodies for lineage markers: anti-Ly6G, anti-CD3e, anti-CD19, anti-B220, anti-CD49b, and anti-Ter119. Lineage^+^ cells were depleted using the MACS system using anti-biotin microbeads. Next, pre-cDCs were sorted as lineage^−^ MHCII^−^ CD172a^int^ CD11c^+^ Flt3^+^ cells as shown in Fig. 4 D. A highly purified population of 1.0 × 10^6^ CD45.2^+^ pre-cDCs was i.v. injected into CD45.1^+^ CD45.2^+^ WT mice. 7 days after the transfer, CD45.1^−^ CD45.2^+^ cells from the SILP and mLNs were analyzed 4 h after flagellin injection.

### Library preparation for mRNA-seq

CD11c^+^ cDCs were enriched from the mLNs of healthy *Il23a*^Venus^ mice using the MACS system, as described above. Subsequently, 1,500 cells of each cDC subset were directly sorted into 500 μl of TRIzol (Thermo Fisher Scientific) using a cell sorter. Total RNA was purified using the Directzol micro prep kit (Zymo Research) and eluted in 8 μl of nuclease-free water. Whole-transcriptome amplification was performed using a modified Quartz-seq2 pipeline (Sasagawa et al., 2018). Briefly, 2 μl of priming buffer (0.48 μl of 2.5 mM dNTPs, 0.25 μl of RNasin plus, 1.3 μl of 0.8333 μM RT primer) was added to 8 μl of purified RNA and subjected to priming reactions at 70°C for 90 s, followed by 35°C for 15 s. Then, 10 μl of RT solution (2 μl of 10× Thermo Pol buffer, 0.25 μl of SuperScriptIII, 0.1375 μl of RNasin Plus, 7.6125 μl of nuclease-free water) was added and the reverse transcription reaction was performed at 4°C for 5 s, 35°C for 5 min, 50°C for 50 min, and 70°C for 15 min. The resultant cDNA was purified using the DNA Clean & Concentrator kit −5 (Zymo Research) and eluted in 9 μl of nuclease-free water. Next, 2.25 μl of poly-A tailing solution (0.625 μl of Thermo Pol buffer, 0.15 μl of 100 mM dATP, 0.12 μl of RNaseH, 0.42 μl of TdT enzyme, 0.935 μl of nuclease-free water) was added, and poly-A tailing reactions were performed at 0°C for 5 s, 37°C for 75 s, and 65°C for 10 min. Then, 46 μl of PCR mix-I (25 μl of 2× Mighty Buffer v2, 0.32 μl of 10 μM tagging primer, 2 μl of Mighty amp Polymerase, 18.8 μl of nuclease-free water) was added, and the second-strand synthesis was performed at 98°C for 130 s, 40°C for 60 s, and heating to 68°C at 0.2°C every second, followed by 68°C for 5 min 50 μl of PCR mix-II (25 μl of 2× Mighty Buffer v2, 0.95 μl of 100 μM PCR primer, 24.2 μl of nuclease-free water) was added, and the cDNA was then amplified for 12 PCR cycles under the following conditions: 98°C for 10 s, 65°C for 15 s, and 68°C for 5 min.

The amplified cDNA was purified using the DNA Clean & Concentrator kit −5, and primer dimers were removed by ×0.65 left side SPRI bead (Beckman Coulter) selection. Illumina DNA libraries were generated using a Nextera XT library preparation kit (Illumina) for sequencing with a NextSeq 500 (Illumina).

The following primers were used for the Quartz-seq2 pipeline: RT primer: 5′-TAT​AGA​ATT​CGC​GGC​CGC​TCG​CGA​TTT​TTT​TTT​TTT​TTT​TTT​TTT​TTT-3′, tagging primer: 5′-TAT​AGA​ATT​CGC​GGC​CGC​TCG​CGA​TAA​TAC​GAC​TCA​CTA​TAG​GGC​GTT​TTT​TTT​TTT​TTT​TTT​TTT​TTT​T-3′, and PCR primer: 5′-(NH2)-GTATAGAATTCGCGGCCGCTCGCGAT-3′.

### Bioinformatic analysis of mRNA-seq data

The sequence reads were mapped to the GRCm39 mouse genome obtained from GENCODE using hisat2 (version 2.1.0; Kim et al., 2019). The read count of each gene was calculated based on the GENCODE gene annotation using FeatureCounts (version 1.6.4; Liao et al., 2014). Transcript per million (TPM) values were then calculated from the read count data. Principal component analysis was carried out using the TPM gene expression matrix. Differential gene expression analysis was performed using DESeq2 (version 1.20.0) to obtain the Log_2_FoldChange, Wald statistic, P value, and adjusted P value (Love et al., 2014).

For the GSEA shown in Fig. 6 C, Notch2 signaling-inducible gene sets in cDC2 subsets were generated from published microarray data (GSE45681; Satpathy et al., 2013). Statistical analysis of the microarray data was carried out using the limma (version 3.38.3) and affy (version 1.60.0) packages (Ritchie et al., 2015; Gautier et al., 2004), and genes with an adjusted P value <0.05 were extracted and ranked by Log_2_FoldChange. The top 100 upregulated genes in splenic CD11b^+^ cDC2 subsets sorted from Notch2^flox/flox^ mice compared with CD11c^Cre^ Notch2^flox/flox^ mice were used.

In Fig. 6 E, 116 genes in category 3 and category 2 that were upregulated by retinoic acid were used as the retinoic acid–inducible gene sets (Balmer and Blomhoff, 2002). All protein-coding genes were sorted by the Wald statistic from DESeq2 comparisons between Il23a-Venus^+^ cDC2s and CD103^+^ CD11b^+^ cDC2s to obtain ranked list files. GSEA was performed with GSEA software (v4.1.0) using these gene sets and the ranked list file, and the enrichment plot, normalized enrichment score (NES), and false discovery rate (FDR) were generated (Subramanian et al., 2005).

### An infection model with *C. rodentium*

The *C. rodentium* DBS100 strain was obtained from the American Type Culture Collection. To prepare for infection, the strain was subcloned and cultured in Luria-Bertani medium for 14 h. The mice were fasted before being orally infected with 2.0 × 10^9^ CFUs of the bacteria. Colon tissues were harvested from the mice 12 days after infection for flow cytometry analysis of cDCs. To evaluate the burden of *C. rodentium*, feces were weighed and homogenized, followed by plating serial dilutions onto MacConkey agar plates (Sigma-Aldrich) for 24 h at 37°C.

### Data presentation and statistical analysis

The data are presented as dot plots from individual mouse with the mean ± standard deviation (SD) displayed for each experimental group. The data are presented as the mean ± standard error of the mean (SEM) in Fig. 6, B and D. Statistical analysis was performed using GraphPad PRISM7, with a two-tailed *t* test, multiple *t* test, or the Mann–Whitney U test for two-group comparisons and one-way ANOVA or two-way ANOVA followed by Tukey’s posttest for grouped data analysis. A P value of <0.05 was considered statistically significant. Sample sizes for all presented data can be found in the figure legends.

### Online supplemental material

Fig. S1 shows screening of surface markers highly correlated with Il23a-Venus expression in gut cDC2s and complements Fig. 2. Fig. S2 amends Fig. 3 and indicates the EpCAM^+^ DCIR2^+^ CD103^−^ CD11b^−^ cDC2s as a primary source of IL-23. Fig. S3 is supplementary to Fig. 5 and shows a surface marker expression pattern of migratory cDCs expressing Il23a-Venus in the mLNs, similar to that of Il23a-Venus^+^ cDCs in the SILP. Fig. S4 shows Il23a-Venus expression in monocytes and macrophages in Notch2^flox/flox^ and CD11c^Cre^ Notch2^flox/flox^ mice during *C. rodentium* infection, as well as the surface marker expression of the remaining Il23a-Venus^+^ cDCs from CD11c^Cre^ Notch2^flox/flox^ mice, related to Fig. 7. Fig. S5 shows the effects of retinoic acid supplementation on the regulation of IL-23–producing cDCs, related to Fig. 8.

## Supplementary Material

SourceData F1 is the source file for Fig. 1.

SourceData F6 is the source file for Fig. 6.

## Data Availability

mRNA-seq data for CD103^+^ CD11b^+^ cDC2s and Il23a-Venus^+^ cDC2s can be found in the DDBJ repository under the accession no. DRA016070.
