# Supplementary material for: Notch2 with retinoic acid license IL-23 expression by intestinal EpCAM+ DCIR2+ cDC2s in mice
Source: J Exp Med. 2024 Jan 5;221(2):e20230923. doi: 10.1084/jem.20230923 (PMC10770806; doi:10.1084/jem.20230923)
Supplement: SourceData F1 — is the source file for Fig. 1. [file JEM_20230923_SourceDataF1.pdf]

SourceData F1

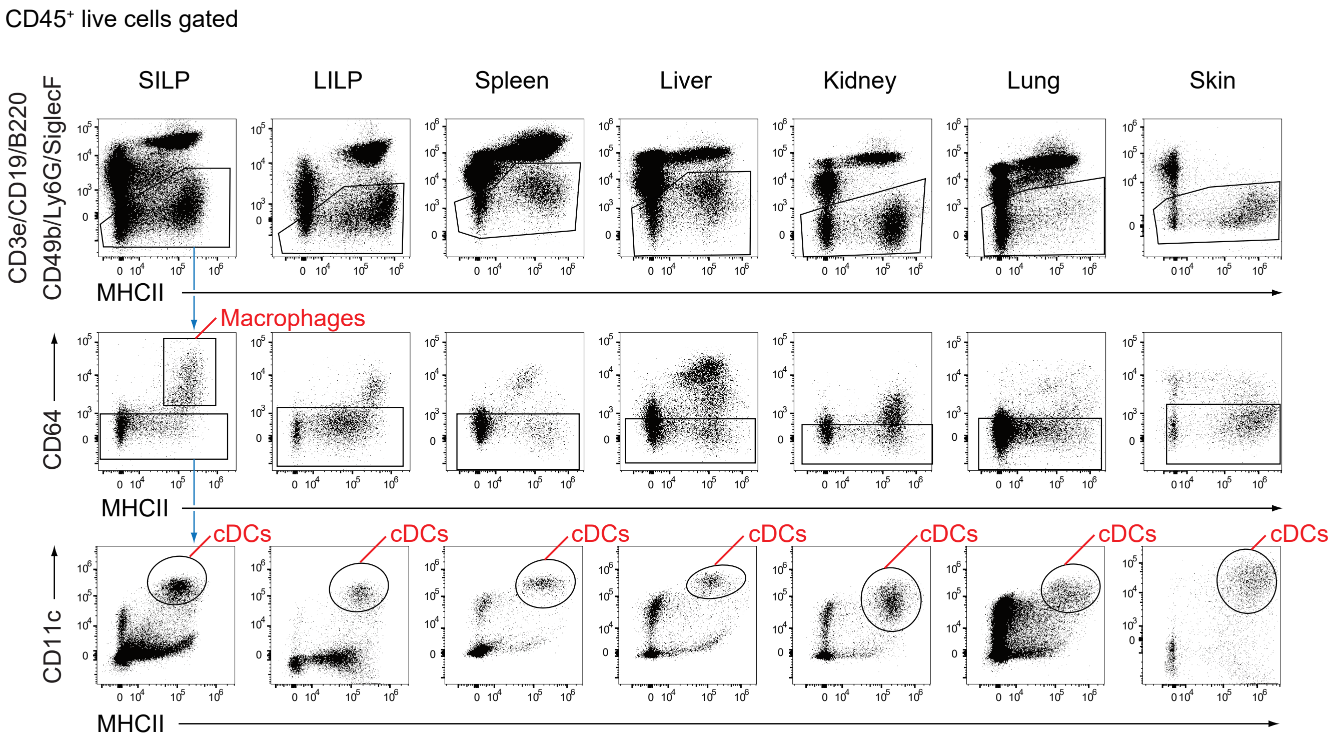

SourceData F1. A gating strategy for defining cDCs and macrophages.

To identify cDCs and macrophages, live $CD45^{+}$ $CD19^{-}$ $B220^{-}$ $CD49b^{-}$ $Ly6G^{-}$ $SiglecF^{-}$ cells in the indicated tissues were gated, followed by gating for $MHCII^{+}$ $CD11c^{+}$ and $MHCII^{+}$ $CD64^{+}$ cells in cDCs and macrophages, respectively.
