# Supplementary material for: Notch2 with retinoic acid license IL-23 expression by intestinal EpCAM+ DCIR2+ cDC2s in mice
Source: J Exp Med. 2024 Jan 5;221(2):e20230923. doi: 10.1084/jem.20230923 (PMC10770806; doi:10.1084/jem.20230923)
Supplement: SourceData F6 — is the source file for Fig. 6. [file JEM_20230923_SourceDataF6.pdf]

# SourceData F6

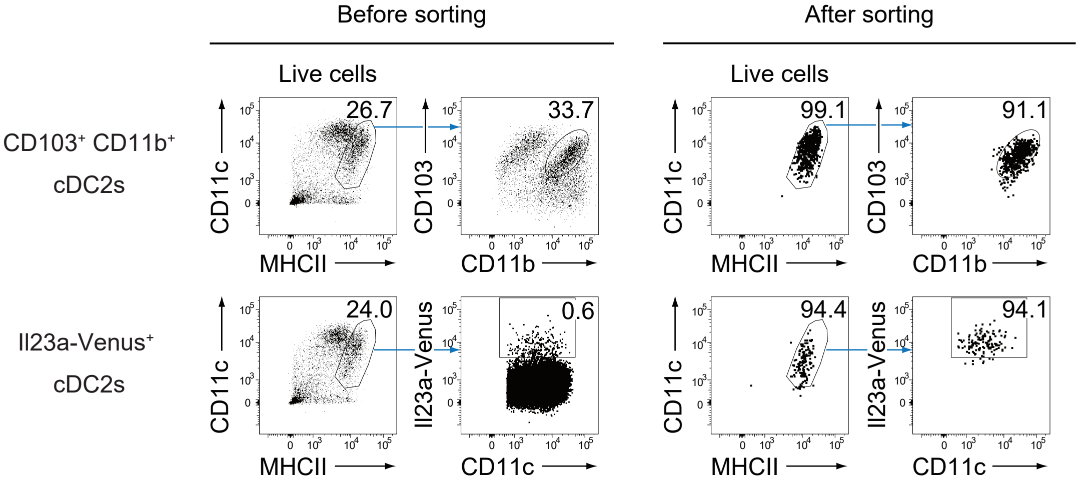


**SourceData F6. The gating strategy for sorting CD103⁺ CD11b⁺ and Il23a-Venus⁺ cDC2s for mRNA-seq analysis.** CD11c⁺ cells from the mLNs of *Il23a*$^{Venus}$ mice were enriched by MACS positive selection with an anti-CD11c antibody. Then, CD103⁺ CD11b⁺ and Il23a-Venus⁺ migratory cDC2s were sorted using the gating strategy depicted in the "Before sorting" panel. The sorting purities were validated and confirmed, as shown in the "After sorting" panel.
